# Interaction between Genetic Predisposition to Adiposity and Dietary Protein in Relation to Subsequent Change in Body Weight and Waist Circumference

**DOI:** 10.1371/journal.pone.0110890

**Published:** 2014-10-28

**Authors:** Mikkel Z. Ankarfeldt, Sofus C. Larsen, Lars Ängquist, Lise Lotte N. Husemoen, Nina Roswall, Kim Overvad, Marianne Uhre Jakobsen, Jytte Halkjær, Anne Tjønneland, Allan Linneberg, Ulla Toft, Torben Hansen, Oluf Pedersen, Berit L. Heitmann, Arne Astrup, Thorkild I. A. Sørensen

**Affiliations:** 1 Institute of Preventive Medicine, Bispebjerg and Frederiksberg Hospital, the Capital Region, Copenhagen, Denmark; 2 Faculty of Medical and Health Sciences, University of Copenhagen, Copenhagen, Denmark; 3 Research Centre for Prevention and Health, Glostrup University Hospital, Copenhagen, Denmark; 4 Danish Cancer Society Research Center, Copenhagen, Denmark; 5 Section for Epidemiology, Department of Public Health, Aarhus University, Aarhus, Denmark; 6 Department of Cardiology, Aalborg University Hospital, Aalborg, Denmark; 7 The Novo Nordisk Foundation Center for Basic Metabolic Research, Section on Metabolic Genetics, Faculty of Health and Medical Sciences, University of Copenhagen, Copenhagen, Denmark; 8 The National Institute of Public Health, University of Southern Denmark, Copenhagen, Denmark; 9 The Boden Institute of Obesity, Nutrition, Exercise & Eating Disorders, University of Sydney, Sydney, Australia; 10 National Institute of Public Health, University of Southern Denmark, Copenhagen, Denmark; 11 Department of Nutrition, Exercise and Sports, NEXS, Faculty of Science, University of Copenhagen, Copenhagen, Denmark; Sudbury Regional Hospital, Canada

## Abstract

**Background:**

Genetic predisposition to adiposity may interact with dietary protein in relation to changes of anthropometry.

**Objective:**

To investigate the interaction between genetic predisposition to higher body mass index (BMI), waist circumference (WC) or waist-hip ratio adjusted for BMI (WHR_BMI_) and dietary protein in relation to subsequent change in body weight (ΔBW) or change in WC (ΔWC).

**Design:**

Three different Danish cohorts were used. In total 7,054 individuals constituted the study population with information on diet, 50 single-nucleotide polymorphisms (SNPs) associated with BMI, WC or WHR_BMI_, as well as potential confounders. Mean follow-up time was ∼5 years. Four genetic predisposition-scores were based on the SNPs; a complete-score including all selected adiposity- associated SNPs, and three scores including BMI, WC or WHR_BMI_ associated polymorphisms, respectively. The association between protein intake and ΔBW or ΔWC were examined and interactions between SNP-score and protein were investigated. Analyses were based on linear regressions using macronutrient substitution models and meta-analyses.

**Results:**

When protein replaced carbohydrate, meta-analyses showed no associations with ΔBW (41.0 gram/y/5 energy% protein, [95% CI: −32.3; 114.3]) or ΔWC (<−0.1 mm/y/5 energy % protein, [−1.1; 1.1]). Similarly, there were no interactions for any SNP-scores and protein for either ΔBW (complete SNP-score: 1.8 gram/y/5 energy% protein/risk allele, [−7.0; 10.6]) or ΔWC (complete SNP-score: <0.1 mm/y/5 energy% protein/risk allele, [−0.1; 0.1]). Similar results were seen when protein replaced fat.

**Conclusion:**

This study indicates that the genetic predisposition to general and abdominal adiposity, assessed by gene-scores, does not seem to modulate the influence of dietary protein on ΔBW or ΔWC**.**

## Introduction

Randomized, controlled trials (RCTs) have shown that among overweight and obese individuals, a high ad libitum protein intake leads to a greater body weight (BW) loss and better weight loss maintenance compared with low protein intake for up to 12 months [1], [2]. When investigated in observational studies greater protein intake is generally associated with weight gain [3], [4], and in some studies also with gain of fat mass [5]. The results from both kinds of studies are based on average weight changes, and show considerable diversity in both directions; thus, individuals with both weight gain and weight loss are present in observational studies as well as in RCTs. The reason behind this diversity, both within studies and across studies, may be genetic differences and gene-diet interactions. Common genetic variants are associated with adiposity measures such as body mass index (BMI), waist circumference (WC) or waist-hip ratio adjusted for BMI (WHR_BMI_) in cross sectional studies [6]–[18]. However, since the adiposity levels in cross-sectional studies must be mixtures of individuals who have remained stable, have increased or have decreased in the particular traits during the preceding time, implications of the genetic variants in individual changes of the traits is possible. It has been found that some individual SNPs may interact with dietary protein in relation to weight change [19]–[22]. Although adiposity-associated SNPs will be most common among overweight and obese individuals, there will be some who do not have these SNPs despite being overweight or obese, as well as there will be normal-weight individuals carrying adiposity-associated SNPs. Therefore, adiposity-associated SNPs and their plausible interaction with dietary protein may be related to for future changes in anthropometry in the general population irrespective of levels of adiposity traits.

We hypothesize that for individuals with several adiposity-associated SNPs, compared to a lower intake, a higher intake of protein will associate with BW loss or BW stability. Contrary, for individuals with few adiposity-associated SNPs, higher intake of protein will be associated with BW gain compared to lower protein intake. The same may be the case for changes in WC, whereby a higher intake of protein will associate with a reduced or stable WC in individuals carrying several adiposity-associated SNPs.

The aim of the present study was therefore to examine the interactions between genetic predisposition to higher BMI, WC or WHR_BMI_ and dietary protein in relation to subsequent long-term change in BW and WC.

## Materials and Methods

The study population was based on three independent Danish cohorts of adults: *MONICA*, Diet, Cancer and Health (*DCH*), and *INTER99*. To be included in the study, information on baseline macronutrient intake, measurements on BW or WC at baseline and follow-up should be available, so that corresponding changes could be calculated, also information on single nucleotide polymorphisms (SNPs) associated with BMI, WC or WHR_BMI_ as well as information on potential confounders should be available.

### The cohorts

#### MONICA

From 11 municipalities in the former Copenhagen County a random sample of 4,581 men and women born in 1922, 1932, 1942 and 1952 were invited, of whom 3,608 participated in a baseline examination during 1982–83. Blood sampling and measures of anthropometrics and dietary intake were performed. A follow-up was conducted during 1987–88. Of 2,987 persons in both the baseline and follow-up examination [23] 1,852 individuals had complete baseline seven-day food records [24], 1,578 in addition had complete information on covariates and measures of BW at both baseline and follow-up, and 1,426 of these also had complete information on genetic variants. For the present study we excluded individuals with prevalent cancer (n = 16), cardiovascular disease (n = 61) or self-reported diabetes (n = 20) at baseline, and individuals with incident cancer (n = 13), cardiovascular disease (n = 57) or self-reported diabetes (n = 2) during follow-up. The final study population consisted of 1,257 individuals.

#### The *DCH* study

Among individuals living in Copenhagen and Aarhus, aged between 50 and 64 years and with no diagnosis of cancer registered in the Danish Cancer Registry, 160,725 were invited to baseline examination during the years 1993–97. A total of 57,053 (35%) accepted the invitation. Measurements of anthropometry were performed and a lifestyle questionnaire and a 192 item semi-quantitative food frequency questionnaire (FFQ) were answered [25]. Follow-up was in 1999–2000 and included self-reported anthropometry. Individuals were included if they were younger than 60 years at baseline and younger than 65 years at follow-up, had no cancer, cardiovascular disease or diabetes at baseline nor developed these diseases during the study period, had stable smoking habits between baseline and follow-up and an average BW gain of no more than 5 kg/year. Among these, individuals from a case-sample of 1,200 BW gainers and a random sample of 1,209 individuals were selected. BW gainers were defined by using the residuals from sex-specific regression models of average BW change per year on baseline values of age, BW, height, smoking status (current/former/never smokers), and follow-up time. The 600 males and the 600 females who experienced the greatest BW gain were selected. The random sample was selected based on the total eligible cohort; a small overlap with individuals also defined as BW gainers (n = 79) were excluded from the final random sample (n = 1,130) [26]. Of the selected 2,330 individuals, information on genes, dietary intake, anthropometrics change and potential confounders was available in 2,167 individuals.

#### *INTER99*

A population-based randomized controlled trial (CT00289237, ClinicalTrials.gov). From 11 municipalities in the former Copenhagen County an age- and sex-stratified random sample of 13,016 individuals born in 1939–40, 1944–45, 1949–50, 1954–55, 1959–60, 1964–65, 1969–70 were invited, of whom 6,784 participated in a baseline examination during 1999–2001. Various blood tests and a physical examination were performed and a self-administered health questionnaire and validated FFQ were completed [27]. The study is further described elsewhere [28], [29]. A follow-up was conducted during 2004–06 where the baseline examination was repeated [30]. Information on diet, genes, baseline and follow-up anthropometric measures and information on potential confounders were available in 4,574 individuals. For the present study, we excluded individuals with prevalent cancer (n = 87), cardiovascular disease (n = 320) or self-reported diabetes (n = 94) at baseline, and individuals with incident cancer (n = 68), cardiovascular disease (n = 256) or self-reported diabetes (n = 119) during follow-up. The final study population consisted of 3,630 individuals.

All procedures were performed in accordance with the Helsinki Declaration, and all participants provided written informed consent in the three cohorts. *MONICA*, *DCH* and *INTER99* have been generated for many other purposes than the present study. Access to the data requires an application submitted to and subsequently approved by the respective Steering Boards of the studies. Contact professor Berit L. Heitmann (Berit.Lilienthal.Heitmann@regionh.dk), professor Anne Tjønneland (annet@cancer.dk) and professor Allan Linneberg (allan.linneberg@regionh.dk) to request to access to MONICA, DCH and INTER99, respectively.

### Anthropometric measurements

In *MONICA*, *DCH* and *INTER99* baseline height was measured to the nearest 0.5 cm and baseline BW to the nearest 0.1 kg. In *MONICA* WC was measured at follow-up but not at baseline, and therefore the *MONICA* cohort was not included in the analyses with WC as outcome. In *DCH* and INTER99 baseline WC was measured to the nearest 1 cm, horizontally midway between the lower rib margin and the iliac crest. Similar procedures were followed in *INTER99* and *MONICA* for follow-up measures of height, BW and WC. In *DCH* the follow-up measures of BW and WC were however self-reported. Instruction to measure the WC at the level of umbilicus and a paper measuring tape were enclosed. *DCH* included a validation study, showing that the Spearman’s correlation coefficients between technician measured and self-reported WC were 0.87 in men and 0.88 in women [31].

Because of variation in follow-up time between cohorts, and between individuals within each cohort, average changes per year in BW and WC were calculated as changes between baseline and follow-up divided by the follow-up time in years for each individual (ΔBW, kg/year; ΔWC, cm/year).

### Assessment of dietary intake

In *MONICA*, participants were instructed verbally and in writing on how to complete a 7-day food record within a three-week period. Information on the average weight of 19 frequently consumed foods (e.g. the weight of a slice of bread, a glass of milk, etc.) was provided. The entries were in grams, estimated or preferably weighted [24].

In *DCH* and in *INTER99* a nearly identical FFQ was completed. The FFQ consisted of 192 and 198 items, respectively, about the average intake of different foods and beverages during the past year. Development and validation of these questionnaires are described elsewhere [27], [32], [33].

The individual, average daily intake of macronutrients and total energy was calculated with the software DANKOST in *MONICA* and with FoodCalc in *DCH* and *INTER99*, both based on the official Danish food composition tables (http://www.foodcomp.dk) [34]–[36].

### Possible confounders

All participants reported smoking habits (never smoked, ex-smokers or current smokers). Information on physical activity was self-reported in all cohorts. In *MONICA* categorized in four groups as: 1) almost completely inactive: sedentary activities such as reading, watching television and going to the movies. 2) Some physical activity: At least 4 hours weekly including for example walking, cycling, constructional work, bowling and table tennis. 3) Regular hard activity at least 3 hours weekly, including for example swimming, tennis and badminton etc. or heavy gardening. 4) Hard activity: Elite sports such as swimming, soccer, badminton or long distance running several times a week. In *DCH* the validated Cambridge Physical Activity Index was calculated by combining occupational physical activity with time spent on cycling and sport in summer and winter [37], and categorized in four groups as: 1) inactive, 2) moderately inactive, 3) moderately active and 4) active. In *INTER99* information on time spent on commuting and leisure time physical activity were used to categorize in four groups as: 1) <2 h/week, 2) 2–<4 h/week, 3) 4–<7 h/week and 4) ≥7 h/week. Information on years of regular schooling was available in all cohorts and categorized as having school education above or below the primary level. Information on age, sex and whether women had entered menopause was also available in all cohorts.

### SNP selection and genotyping

Through review of genome-wide association studies (GWAS) published until 2010, 58 SNPs were identified to be consistently associated with BMI, WC or WHR_BMI_ [6]–[18] of which 50 SNPs were available in all the three cohorts in the present study (Table S1 in File S1).

In *MONICA* and *DCH* the SNPs were genotyped with the KASPar SNP genotyping method (KBioscience, Hoddesdon, UK). In *MONICA* the average genotyping success rate was 98.3% (minimum 95.8%). In *DCH* the average genotyping success rate was 97.8% and 185 replicate samples had a success rate above 98% and an error rate below 0.5%. The number of individuals with available SNP information in *DCH* was a little different, since only information on rs9939609 of FTO were available in all individuals (n = 2,167), while information on other SNPs was a little lower (n = 1,849–1,885).

In *INTER99* the SNPs were successfully genotyped using either the KASPar method (48 SNPs), or through Human Cardio-metabo bead chip array (2 SNPs; rs7138803 and rs7647305) using Illumina Hi-Scan technology and GenomeStudio software (http://www.illumina.com/systems/hiscan.ilmn) and had an average genotyping success rate of 96.7% (minimum: 94.7).

### Genetic predisposition-scores

For each individual, the 50 SNPs were coded 0, 1 or 2 according to number of adiposity-associated risk alleles. Genetic predisposition to adiposity was illustrated through four SNP-scores by summing up corresponding risk alleles for each participant; a complete-score consisting of all 50 SNPs (individuals with information on score, n: *MONICA* = 836, *DCH* = 1,247, *INTER99* = 1,844), and three phenotype-specific scores in order to reduce random variation, one based on the subset of 33 BMI associated SNPs (n: *MONICA* = 941, *DCH* = 1,438, *INTER99* = 2,222), one on the six WC associated SNPs (n: *MONICA* = 1,185, *DCH* = 1,805, *INTER99* = 3,010) and one on the 14 WHR_BMI_ associated SNPs (n: *MONICA* = 1,121, *DCH* = 1,624, *INTER99* = 2,913). Higher scores indicate higher genetic predisposition.

### Statistical analyses

The analyses of the association between ΔBW or ΔWC and dietary protein, each of the four SNP-scores, as well as the interaction of dietary protein and the four SNP-scores were based on multiple linear regressions. Macronutrients energy substitution models were used, as described below. Adjustments were done for the possible confounders described above and for the baseline level of the outcome measure. To investigate the association with ΔWC independently of ΔBW, the analyses of ΔWC were also adjusted for ΔBW.

Both fixed- and random effect meta-analyses were performed in order to produce combined results over the three cohorts. The inverse variance approach was used to weight the study-specific effect-estimates [38], [39]. To assess the heterogeneity between the cohorts, I^2^ values - indicating the amount of total variation explained by between-study variation [40] - and Q tests were used. I^2^ values of 0–25%, 25–50%, 50–75% and 75–100% were considered showing no, moderate, significant and extreme heterogeneity, respectively [41]. Since some heterogeneity was present only the results from the random effect analyses are shown.

The *DCH* study is based on both a random sample and a sample of BW gainers, but interaction analyses of such subgroup effects were non-significant, and therefore not included in the reported results. Similarly, *INTER99* is a multi-factorial lifestyle intervention study, with individual and group counselling focussing on smoking, physical activity, diet and alcohol as the intervention, and adjustment for baseline intervention did not notably differ from the reported results.

Exploratory interaction analyses based on individual SNPs and dietary protein intake were also run using multiple linear regressions, with adjustment as above, and adjusted for multiple testing through Bonferroni correction of the 50 tests performed.

A threshold of p-values ≤0.05 was used to declare statistical significance. The analyses were performed with Stata 12.1 (StataCorp, College Station, Texas, USA; www.stata.com).

### Macronutrient substitution models

The analyses used macronutrients energy substitution models [42] to investigate the associations when protein replaced either fat or carbohydrate. The implementation depends on the total energy intake being distributed into four parts – the macronutrients protein, fat, carbohydrates and alcohol – and that the relative energy intake (E%) of one macronutrient can be estimated if the E% of all the other macronutrients are known. Hence, our substitution models are then parameterized by including three out of four macronutrients into each regression model, i.e. E% of protein, alcohol and either fat or carbohydrate intake. The estimated corresponding regression coefficients can then be interpreted as the effect of 1 E% protein replacing 1 E% of the omitted macronutrient since the E% of the other two macronutrients is assumed constant [42]. Greater protein intake might decrease the total energy intake. Hence the substitution models did not adjust for total energy intake, and should be interpreted as changing the relative intake of protein, E%, with possible change of total energy intake as well. However, we also wanted to investigate if there is an interaction between genetic predisposition to adiposity and the effect of protein replacing another macronutrient. Adding a standard product-based interaction term (genetic predisposition × protein) would not suffice to capture such interaction-effects with respect to the actual substitution (but rather with respect to protein in itself, i.e. the outcome will then be independent of which macronutrient protein is replacing). Hence, the macronutrient energy substitution model was re-parameterized to include a single variable describing the substitution (protein replacing either fat or carbohydrate) as well as the two variables for the macronutrients not being replaced (fat or carbohydrate, and alcohol). The substitution parameter equals half of the difference of protein and the macronutrient that is replaced (as e.g. [E% protein-E% fat]/2). The interpretation then remains the same as above, and one can also directly use this single variable in order to create, and to test for, true substitution interaction effects. Further details are found in File S1.

### Supplementary analysis

As a supplementary analysis, we also investigated if the protein intake depended on energy balance and genetic predisposition to adiposity. Since it is impossible to determine energy balance as a difference in energy intake and energy expenditure over the observation period, we used the observed body weight changes as a presumed direct measure of the cumulative net energy balance over the entire observation period. We divided the participants in three groups according to weight change during the study period. Within each of the three cohorts, participants with an average weight loss of >0.5 kg/year were considered having a negative energy balance, participants with an average weight change of ±0.5 kg/year were considered having a neutral energy balance, and participants with an average weight gain of >0.5 kg/year were considered having a positive energy balance. Each of the three energy balance groups was further divided according to whether they had a BMI-associated SNP-score above or below the mean. For each of these six groups we estimated the average protein intake both as E% and as gram/kg body weight/day, as well as the SEM and derived 95% confidence intervals, and these estimates were then compared across the six groups.

## Results

In total, 7,054 individuals were included in the present study. Of these, 1,257 were from *MONICA*, 2,167 were from *DCH* and 3,630 were from *INTER99*. **Table 1** shows descriptive information of anthropometrics, dietary intake, SNP-scores and covariates in the three cohorts. In *DCH*, ΔBW and ΔWC were greater than for the other cohorts, probably reflecting the case-cohort design. The median intake of protein in *DCH MONICA* and *INTER99* ranged from 14.0–17.7% of energy intake. The three cohorts were similar with regard to median, 5- and 95 percentiles of the genetic predisposition-scores.

**Table 1:** Information on dietary intake, covariates, anthropometrics and genetic predisposition-scores.

	MONICA	DCH	INTER99
N	1,257	2,167	3,630
Follow-up time (years)	5.0 (4.9; 5.3)	5.3 (5.0; 5.8)	5.4 (5.1; 5.7)
**Dietary variables**			
Protein intake (E%)	14.0 (10.3; 19.7)	17.7 (13.6; 22.4)	14.8 (11.4; 19.0)
Carbohydrate intake (E%)	35.9 (26.1; 47.2)	41.6 (31.8; 52.9)	47.5 (35.6; 61.0)
Fat intake (E%)	44.2 (32.9; 54.3)	34.2 (24.6; 42.7)	32.4 (21.6; 43.9)
Alcohol intake (E%)	4.1 (0.0; 17.7)	4.3 (0.2; 19.2)	3.0 (0.0; 14.9)
Energy (MJ/d)	9.0 (5.1; 14.9)	8.8 (5.3; 14.2)	9.4 (5.2; 15.8)
**Covariates**			
Baseline age (years)	41.4 (30.6; 61.1)	53.0 (50.0; 58.0)	45.0 (34.7; 59.8)
Height (cm)	169 (156; 184)	171 (158; 186)	172 (158; 188)
Sex, % women	52.1	49.4	51.7
Smoking, % Never smokers	27.6	41.4	40.7
Education, % ≤Primary school	34.5	30.1	25.0
Physical activity, % most sedentary group	21.6	9.5	11.1
Menopausal status, % postmenopausal	41.7	55.6	25.6
**BW (kg)**			
At baseline (kg)	69.0 (52.0; 93.0)	77.1 (56.8; 104.4)	75.1 (54.8; 102.3)
At follow-up (kg)	69.9 (51.4; 94.7)	82.0 (58.0; 110.0)	76.0 (55.5; 103.5)
ΔBW (kg/year)	0.24 (−0.98; 1.62)	1.02 (−1.05; 2.39)	0.18 (−1.40; 1.70)
**WC (cm)** 1			
At baseline (kg)	-	90 (70; 112)	84 (67; 105)
At follow-up (kg)	-	98 (76; 121)	87 (69; 109)
ΔWC (cm/year)	-	1.37 (−0.78; 4.41)	0.54 (−1.27; 2.46)
**SNP-based variables** 2			
BMI SNP-score	29 (23; 35)	28 (23; 35)	29 (23; 34)
WC SNP-score	3 (1; 6)	3 (1; 6)	3 (1; 6)
WHR_BMI_ SNP-score	14 (10; 18)	14 (10; 18)	14 (10; 18)
Complete SNP-score	44 (37; 52)	44 (37; 51)	44 (36; 51)

Abbreviations: BMI, body-mass index. BW, body weight; E%, percentage of total energy intake; WC, waist circumference; WHR_BMI_, waist-hip-ratio adjusted for BMI. ΔBW, annual weight change; ΔWC, annual WC change.

1 In *MONICA* information was not available. In *DCH* n = 2,165 with baseline WC, n = 2,130 with follow-up WC and n = 2,128 with ΔWC. In *INTER99* n = 3,626 with baseline WC, n = 3,163 with follow-up WC and n = 3,160 with ΔWC.

2 Sum of BMI, WC or WHR_BMI_ associated risk-alleles. In *MONICA* information was available in n = 941 with BMI SNP-score, n = 1,185 with WC SNP-score, n = 1,121 with WHR_BMI_ SNP-score and n = 836 with complete SNP-score. In *DCH* n = 1,438 with BMI SNP-score, n = 1,805 with WC SNP-score, n = 1,624 with WHR_BMI_ SNP-score and n = 1,247 with complete SNP-score. In *INTER99* n = 2,222 with BMI SNP-score, n = 3,010 with WC SNP-score, n = 2,913 with WHR_BMI_ SNP-score, n = 1,844 with complete-score.

Reported as median (5–95 percentiles) unless otherwise stated.

**Table S1** in File S1 includes the information on the individual SNPs and the adiposity traits they are associated with.

**Table 2** presents the associations between dietary protein and ΔBW and ΔWC adjusted for potential confounders, when protein replaced either fat or carbohydrate. In *MONICA,* a significant association was seen between dietary protein and weight gain, when replacing carbohydrate. A similar, but non-significant, tendency was seen when dietary protein replaced fat. However, the meta-analyses showed no overall significant associations across any substitution models or outcome measures.

**Table 2:** Change of body weight or waist circumference pr. 5 energy % protein replacing carbohydrate or fat.

	Change in body weight (gram/year)			Change in waist circumference (mm/year)		
	N	Adjusted β (95% CI)^1^	% weight^2^	N	Adjusted β (95% CI)^1^	% weight^2^
**Protein replacing carbohydrate**						
MONICA	1,257	123.4 (30.4; 216.4)	30.04	-	-	-
DCH	2,167	−11.6 (−102.6; 79.5)	30.65	2,128	−0.7 (−1.8; 0.4)	41.66
INTER99	3,630	19.0 (−47.6; 85.6)	39.31	3,160	0.5 (−0.1; 1.0)	58.34
Overall	7,054	41.0 (−32.3; 114.3)	100.00	5,327	<−0.1 (−1.1; 1.1)	100.00
**Protein replacing fat**						
MONICA	1,257	56.3 (−31.9; 144.4)	29.41	-	-	-
DCH	2,167	54.5 (−45.1; 154.1)	23.01	2,167	−0.8 (−2.0; 0.4)	41.50
INTER99	3,630	15.2 (−54.1; 84.5)	47.58	3,160	0.5 (−0.1; 1.1)	58.50
Overall	7,054	36.3 (−11.5; 84.1)	100.00	5,327	−0.1 (−1.3; 1.2)	100.00

As the four SNP-scores are based on SNPs showing association with adiposity traits, statistically significant associations with baseline level of BW and WC were seen, but not with the WHR_BMI_-score. There were, however, no statistically significant associations between any of the SNP-scores and ΔBW or ΔWC. These analyses are reported elsewhere [43].

**Figure 1:**
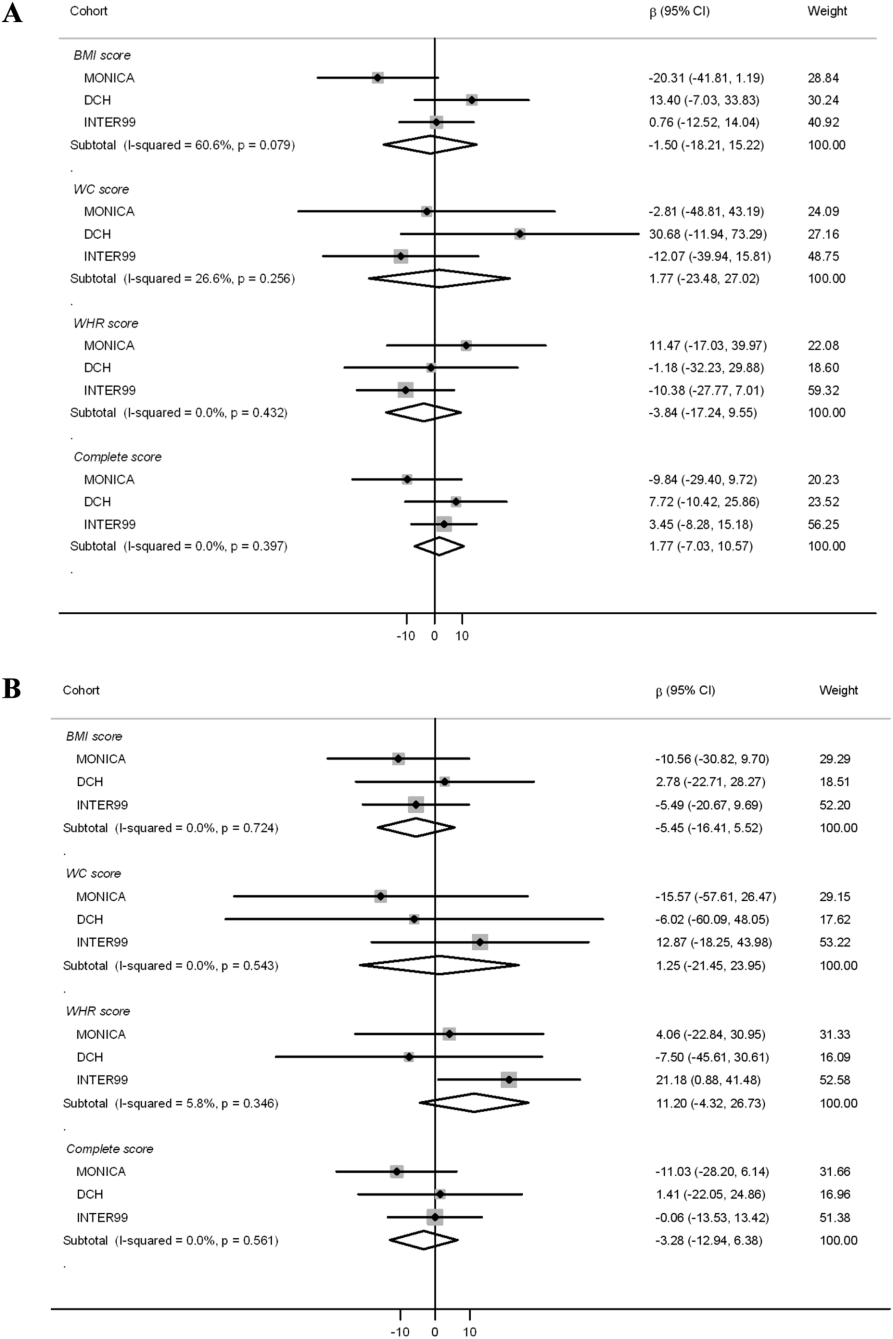
Interaction of SNP-scores and protein intake. Average, yearly weight change (gram/y/5 E% protein/risk allele). A: protein replacing carbohydrate. B: protein replacing fat. SNP-score×protein interactions were calculated using linear regression and pooled estimates were calculated using random effect meta-analysis. The effect-estimates from the individual cohorts were weighted by the inverses of their variance (% weight). The results were adjusted for baseline weight, height, sex, smoking status, physical activity, education and menopausal status for women. Abbreviations: BMI-score, sum of body mass index associated risk-alleles; WC-score, sum of waist circumference associated risk-alleles; WHR_BMI_-score, sum of waist-hip ratio adjusted for BMI associated risk-alleles.

**Figure 2:**
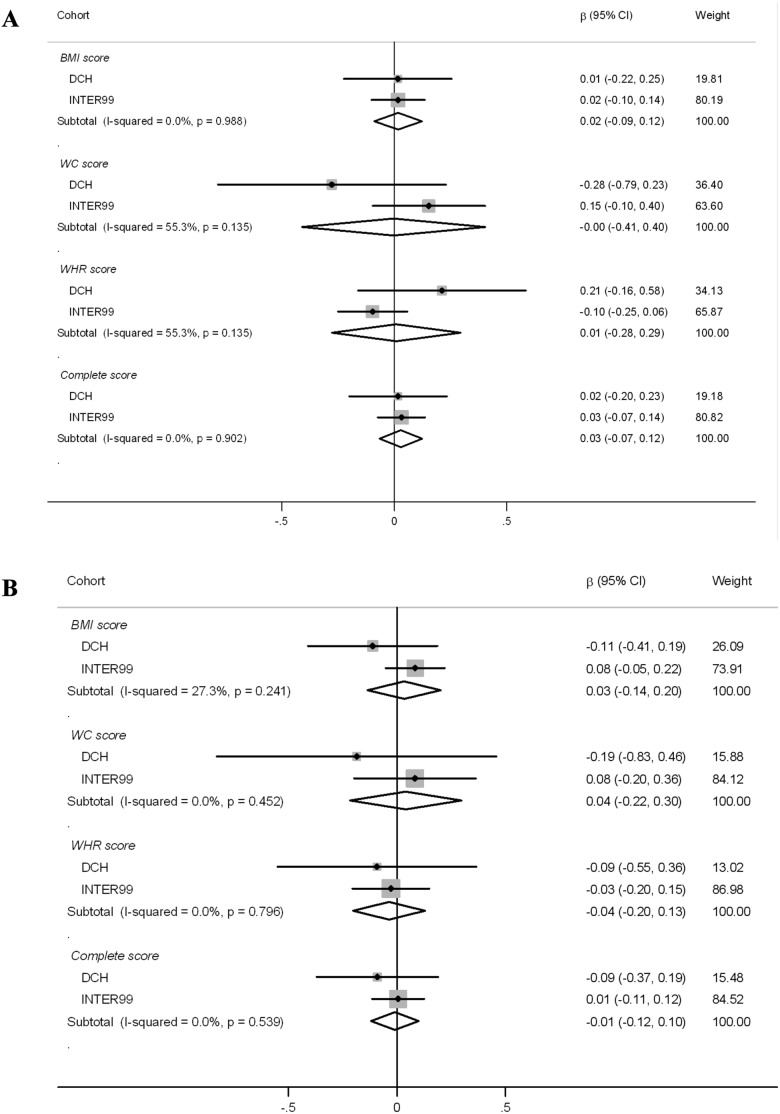
Interaction of SNP-scores and protein intake. Average, yearly waist change (mm/y/5 E% protein/risk allele). A: protein replacing carbohydrate. B: protein replacing fat. SNP-score×protein interactions were calculated using linear regression and pooled estimates were calculated using random effect meta-analysis. The effect-estimates from the individual cohorts were weighted by the inverses of their variance (% weight). The results were adjusted for baseline waist circumference, height, sex, smoking status, physical activity, education, menopausal status for women and concurrent change in body weight. Abbreviations: BMI-score, sum of body mass index associated risk-alleles; WC-score, sum of waist circumference associated risk-alleles; WHR_BMI_-score, sum of waist-hip ratio adjusted for BMI associated risk-alleles.

Interactions between the three phenotype-specific scores or the complete SNP-score and dietary protein in relation to ΔBW and ΔWC are presented in **Figure 1** and **Figure 2** (Figure 1A–B: ΔBW, Figure 2A–B: ΔWC). Overall, the meta-analyses showed no interactions for any of the SNP-scores, whether protein replaced carbohydrate or fat.

Finally, for exploratory purposes, the interaction between the individual SNPs and dietary protein in relation to ΔBW and ΔWC were investigated (**Table S2** in File S1). In the *DCH*, cohort a single SNP (rs6602024 of PFKP) interacted significantly with dietary protein in relation to ΔBW, when replacing fat (β = −328 gram/y/5 E% protein/risk allele, corrected p = 0.022), and in *MONICA* a single SNP (rs2568958 of NEGR1) interacted significantly with dietary protein in relation to ΔBW, when replacing carbohydrate (β = −167 gram/y/5 E% protein/risk allele, corrected p = 0.045). None of these were seen in the other cohorts.

The results from the supplementary analysis of protein intake in subgroups of participants with high or low SNP-score and positive, neutral and negative net cumulative energy balance as indicated by weight changes are shown in **Table S3** in File S1. There were no consistent differences in protein intake between groups with high or low SNP-score or negative, neutral or positive energy balance.

## Discussion

Interactions between dietary protein, genetic predisposition-scores and ΔBW and ΔWC were investigated in meta-analyses of three Danish cohorts. Overall, no interactions were seen for any SNP-scores, substitution models or outcome measures. These null findings are trustworthy, as the confidence intervals were narrow, indicating that the true values that could possibly generate the observed results are small, very close to zero and of limited importance. As an example, the point estimate for interaction between dietary protein replacing carbohydrate and the WHR_BMI_-score in relation to ΔBW is −3.84 grams/y/5 E % protein/risk allele (non-significant, Figure 1A); Even if replacing additional 5 E% of carbohydrate with protein (The habitual diet = 12–15 E%) and adding as many as 20 risk alleles (overall median = 14) the estimated ΔBW interaction effect is still only 76.8 grams pr. year.

Generally, studies on gene-diet interaction have shown relatively weak association.The study population of 7,054 individuals might be too small to discover a potential interaction between genetic predisposition to adiposity and dietary protein. On the other hand, the narrow confidence intervals suggest sufficient statistical power, and that the null-findings are reliable.

SNP-scores were used to increase power compared with the use of individuals SNPs, and other studies have successfully investigated the interaction of genes and environment in relation to adiposity using similar approaches [44]–[46]. On the other hand, the use of SNP-scores is not always straightforward. The investigated interactions between dietary protein and the genetic predisposition were based on four predisposition-scores, defined as the sum of risk alleles from 50 SNPs associated with BMI, WC and WHR_BMI_, respectively, either all together or individually. This involves the assumption, that the direction of the main effects of the individual SNPs are consistent with the directions of the interaction between the SNP and dietary protein. Such an assumption may be too simplistic. The SNPs were selected based on associations found in cross-sectional analyses, and have not as such been associated with changes in the same traits [43]. However, associations with changes in the traits would require changes in the behaviour or the environment, and this could well be changes in the diet. Thus, it is relevant to investigate prospectively if changes in the traits are associated with interactions with dietary factors such as protein content, which, if associated with subsequent changes in adiposity traits, can be presumed to have changed until the baseline assessment. Although the SNPs were selected from studies of specific adiposity traits, their mutual relationships and the uncertainty about involvement in changes justified analyses of both ΔBW and ΔWC as outcome were performed throughout.

The present study also included main effect analyses of protein and SNPs, respectively. No associations between dietary protein or SNP-scores and ΔBW or ΔWC were found. The exploratory interaction analyses between dietary protein and the individual SNPs showed two suggestive cohort-specific interactions in relation to ΔBW (in *DCH*: rs6602024, nearest gene: PFKP; in *MONICA*: rs2568958, nearest gene: NEGR1). This was however not replicated in the other cohorts, and therefore interpreted as significance by chance. Although interactions between dietary protein and *FTO* or *TFAP2B*-variations in relation with weight change have previously been found [20], [21], such interactions were not detected in the present study.

Previous experimental studies have indicated that the effect of dietary protein on body weight regulation may depend on the energy balance [47]. Since measuring energy intake and energy expenditure and monitoring these components during the observation period with any acceptable precision and accuracy is impossible, we used the simplistic approach assuming that the body weight change is a direct measure of the net cumulative energy balance. We found no relevant differences in protein intake between the subgroups with different energy balance and different SNP-scores. This suggests that energy balance is not important for the interaction between dietary protein and SNP-score level in the effect on subsequent weight changes. Future studies on this should have both greater statistical power and use measures of changes in energy-carrying compartments of the body composition as a reflection of the net cumulative energy balance.

The strengths of the present study are the detailed information on dietary intake, measured either by 7-day food records or validated FFQs, the information of the genetic predisposition to adiposity based on 50 SNPs consistently associated with adiposity traits, the information on BW and WC measured at baseline and follow-up, as well as information on potential confounders.

Limitations to the present study include, that even though measures of dietary intake are validated, both 7-day food records and FFQs are subject to some inaccuracies. Urinary nitrogen is a biomarker for protein intake, and the use of such objective measure would have strengthened the present study. The protein intake in the included cohorts reflects the range of a habitual diet, while many RCTs, reporting weight loss and maintenance to be associated with a high protein diet, investigate levels of dietary protein that are considerably higher than a habitual diet (20–35 E%). It is possible that higher levels of protein intake than in the current cohorts are needed to detect interactions with genetic predisposition to adiposity. As in every epidemiological study, drop-out from baseline to follow-up may imply some selection bias, although we have no reason to suspect it affects the overall findings. The three cohorts in the present study differ with regard to the enrolled participants and the design of the study. This might have affected the possibility to replicate findings across cohorts, since similar design and population are often warranted. The study deals only with adults, and the importance of genetic predisposition and dietary protein in relation to obesity may be different in infants and children due to the implications of growth requirements. The study is based on the assumption that the level of protein intake at baseline is an indication of a departure from the protein intake that would be associated with stable adiposity levels. It is a limitation of the study that information of preceding or subsequent protein intake preceding the observed changes in adiposity levels is not available, which would require a closer monitoring of the dietary intake than what seems feasible in the general population setting. Inclusion of protein intake at the time of follow-up assessment of the changes in adiposity levels would not help in interpretation of the findings because of the likely influences of current level and recent changes in adiposity on the actual as well as reported dietary intake; differences in adiposity traits may be associated with concomitant differences in energy needs and the current adiposity level may be accompanied by the individuals’ wish to oppose the adiposity level as well as a temptation to provide a biased reporting of the intake (desirability bias).

The conflict between the results from observational population-based studies and RCTs investigating the relation between dietary protein and weight change may be due to other differences than genetic predisposition to adiposity. The studies differ in several ways, including intervention context vs. free-living, levels and composition of protein intake, change of protein intake, length of follow-up and risk of confounding. Consequently, the studies and the results may not be directly comparable as also discussed elsewhere [48].

In conclusion, the relation of habitual dietary protein and subsequent ΔBW or ΔWC and the interaction of genetic predisposition to adiposity, based on scores of the selected adiposity-related SNPs, were investigated in three Danish cohorts by meta-analyses. Overall, no associations between dietary protein and ΔBW or ΔWC, as well as no interactions between dietary protein and genetic predisposition were seen. The narrow confidence intervals indicate that most likely no corresponding noteworthy gene-diet interactions, among those addressed here, are present.

## Supporting Information

File S1 **Table S1.** Information on the 50 SNPs included in the study. The individual SNPs are sorted by refSNP (rs) number and grouped according to their associated trait. **Table S2.** Interaction between 50 adiposity-related SNPs and dietary protein replacing either carbohydrate or fat in relation to change in BW or WC in the three cohorts. Corrected p-value: Adjusting for multiple testing through Bonferroni correction of the 50 test performed. **Table S3.** Protein intake within energy balance and BMI SNP-score level.
(DOCX)
