# Supplementary material for: Interaction between Genetic Predisposition to Adiposity and Dietary Protein in Relation to Subsequent Change in Body Weight and Waist Circumference
Source: PLoS One. 2014 Oct 28;9(10):e110890. doi: 10.1371/journal.pone.0110890 (PMC4211714; doi:10.1371/journal.pone.0110890)
Supplement: File S1 — Table S1. Information on the 50 SNPs included in the study. The individual SNPs are sorted by refSNP (rs) number and grouped according to their associated trait. Table S2. Interaction between 50 adiposity-related SNPs and dietary protein replacing either carbohydrate or fat in relation to change in BW or WC in the three cohorts. Corrected p-value: Adjusting for multiple testing through Bonferroni correction of the 50 test performed. Table S3. Protein intake within energy balance and BMI SNP-score level. (DOCX) [file pone.0110890.s001.docx]

**File S1**: Supplementary Material.

**Interaction between genetic predisposition to adiposity and dietary protein in relation to subsequent change in body weight and waist circumference**

Mikkel Z Ankarfeldt^1,2^, Sofus C Larsen^1,2^, Lars Ängquist^1^, Lise Lotte N. Husemoen^3^, Nina Roswall^4^, Kim Overvad^5, 6^, Marianne Uhre Jakobsen^5^, Jytte Halkjær^4^, Anne Tjønneland^4^, Allan Linneberg^3^, Ulla Toft^3^, Torben Hansen^7^, Oluf Pedersen^7^, Berit L Heitmann^1,8,9,10^, Arne Astrup^11^, Thorkild I.A. Sørensen^1, 7^

^1^ Institute of Preventive Medicine, Bispebjerg and Frederiksberg Hospital, the Capital Region, Copenhagen, Denmark

^2^Faculty of Medical and Health Sciences, University of Copenhagen, Copenhagen, Denmark

^3^Research Centre for Prevention and Health, Glostrup University Hospital, Denmark

^4^Danish Cancer Society Research Center, Copenhagen, Denmark

^5^Section for Epidemiology, Department of Public Health, Aarhus University, Denmark

^6^Department of Cardiology, Aalborg University Hospital, Aalborg, Denmark

^7^The Novo Nordisk Foundation Center for Basic Metabolic Research, Section on Metabolic Genetics, Faculty of Health and Medical Sciences, University of Copenhagen, Denmark

^8^The National Institute of Public Health, University of Southern Denmark, Copenhagen, Denmark

^9^The Boden Institute of Obesity, Nutrition, Exercise & Eating Disorders, University of Sydney, Australia

^10^National Institute of Public Health, University of Southern Denmark, Denmark

^11^Department of Nutrition, Exercise and Sports, NEXS, Faculty of Science, University of Copenhagen**Note: Nutritional substitution models**

Below the energy-based basic macronutrient substitution model and the corresponding extended substitution-interaction model are described. For overview and general information on nutritional modeling, see e.g. the seminal textbook Willett (2013); an application of nutritional substitution regression models is given in, as an example, Saldana et al. (2004).

Basic Nutritional Models

Assume an outcome variable *O*, and macronutrient intake-covariates protein (*P*), fat (*F*), carbohydrate (*C*) and alcohol (*A*) here measured as absolute energy intakes; by construction the four parts sum up to a total energy intake (*EI*).

Let us first consider the standard linear regression model

*O = β_0_ + β_P_P + β_F_F + β_C_C + β_A_A + · · ·* (1)

which is a so called energy-partition model where all four macronutrients are entered as explanatory variables. Now, if removing one of the four components from the model, say carbohydrates, and include total energy intake, the model will be slightly modified into

*O = β_0_ + β_P_P + β_F_F + β_A_A + β_EI_EI + · · ·*  (2)

which instead is an energy substitution model with the interpretation that the effect fitted ­–for instance with respect to protein intake, *β_P_* – is the effect of replacing 1 unit of carbohydrates with 1 unit of protein rather than the simple effect of increasing the actual protein intake with 1 unit.

One may note that (1) and (2) are related through the fitted effects through the coefficient constraint

*β_P_ ← [β_P_ − β_C_]* ,

i.e. in the latter (substitution) case the coefficient is bound to equal the difference between the coefficients corresponding to the two substitution-factors from the former (partition) model. Further, the substitutions here of course relate to pseudo-substitutions since one doesn’t really investigate *true* substitutions, but rather compares hypothetical individuals being equal in all senses but the to-be-interpreted ones, based on the derived prediction model.

Basic Substitution-Interaction Models

Next, let us now generalize (2) to include an interaction,

*O = β_0_ + β_P_P + β_P×I_P×I + β_I_I + β_F_F + β_A_A + β_EI_EI + · · ·* (3)

where *I* is an indicator variable, *P×I* is a standard product-based interaction variable and *β_P×I_* refers to the corresponding (indicator variable) protein interaction effect.

Though the main substitution-effect, *β_P,_* in (3), will differ based on which dietary factor to be replacing the corresponding interaction effect, *β_P×I_* in (3), will not depend on this factor. Hence results based on the interaction covariate alone will here be equivalent irrespective of whether protein is in fact modelled to be replacing fat, carbohydrates or alcohol.

Basic Two-Part Substitution-Interaction Models

Now, if wanting the interaction effect to potentially depend on the macronutrient that is to be replaced – to allow for what might be called a two-part interaction – one may simply add a second interaction covariate (related this time to the replaced macronutrient)

*O = β_0_ + β_P_P + β_P×I_P×I + β_C×I_C×I + β_I_I + β_F_F + β_A_A + β_EI_EI + · · ·* (4)

which in our example-case relates to carbohydrates; leading to fitted substitution-effects over the categories of the indicator variable as

*β_P_ (I=0)*

*β_P_ + [β_PxI_ – β_CxI_] (I=1)*

Hence the effect related to a corresponding interaction test would be measured by the estimated implicit expression *[β_P×I_ – β_C×I_]*, which clearly depends also on the *to-be-substituted* factor. The main drawback of this model is then that the actual substitution-interaction depends on two parameters corresponding to two distinct variables and hence makes interpretations less immediate and straightforward. (One may also note that (4) may equally well be equivalently expressed as an extended partition model.)

An Alternative Two-Part Substitution-Interaction Model

An alternative model-parameterization that could potentially ease the derivation of two-part effects (through transforming this estimated effect to depend on a single parameter) might, for example, be formulated as a *half-difference* substitution model (here first considering the nutrient only case):

*O = β_0_ + β_PC_PC + β_F_F + β_A_A + β_EI_EI + · · ·*  (5)

where *PC =[P-C]/2* is half the difference between the two corresponding macronutrients, protein and carbohydrates. Conditioning on the other variables in (5), the interpretation of a 1-unit increase of *PC* would then correspond to a replacement of 1-unit carbohydrates with 1-unit protein. (Reversing the argument, increasing *P* with 1 unit and decreasing *C* with 1 unit will lead to an increase of *(P-C)* with 2 units, hence *PC=(P-C)/2* will then increase with 1 unit.)

Following this, a two-part interaction substitution model can be formulated (re-parameterized) as:

*O = β_0_ + β_PC_PC + β_PC×I_PC×I + β_I_I + β_F_F + β_A_A + β_EI_EI + · · ·*  (6)

where the single regression coefficient, *β_PC×I_*, describes the absolute two-part substitution-interaction effect; further, the joint substitution effect is then described by

*β_PC_ (I=0)*

*β_PC_ + β_PC×I_ (I=1)*

Example: Our Implementation and Application

In our specific case, instead of forming interaction based on indicator variables, we base them on continuous SNP-score variables, summing up corresponding risk alleles. (Implicit in this construction of SNP-scores is the assumption of additive genetic effects). For the analyses, we used the half-difference substitution approach based on (5) and (6) for the pure nutrient and nutrient-interaction substitution-model cases, respectively. Given this setting, estimated effects will then be related to the (average) effect of the presence of one further risk allele.

Also, slightly different from the substitution model outlined above, we used relative macronutrient energy-intakes (% of *EI*) instead of absolute intakes. Since the parts then sum up to a constant, *P + F + C + A = 100 %,* one can directly derive the relative intake of one macronutrient if knowing the intakes of the other three. Therefor the above explanation of the substitution model still overall holds true, while the variable of total energy intake is in fact not needed in the models in order to achieve substitution-interpretations – though it may still be included in order to adjust for the total absolute energy intake. Generally, similar models (i.e. substitution model-structures) may formally be set-up in all cases where several parts are summing up to some kind of a total.

References

Saldana, T. M., Siega-Riz, A. M., & Adair, L. S. (2004). Effect of macronutrient intake on the development of glucose intolerance during pregnancy. American Journal of Clinical Nutrition, 79 (3), 479-486.

Willett, W. (2013). Nutritional epidemiology (third ed.). New York: Oxford University Press.

**Table S1:** Information on the 50 adiposity-related SNPs included in the study. The individual SNPs are sorted by refSNP (rs) number and grouped according to their associated trait.

| **Trait** | **SNP** | **Nearest gene** | **Risk allele** | **Other allele** |
| --- | --- | --- | --- | --- |
|  |  |  |  |  |
| BMI | rs10508503 | *PTER* | C | T |
| BMI | rs10838738 | *MTCH2* | G | A |
| BMI | rs10938397 | *GNPDA2* | G | A |
| BMI | rs10968576 | *LRRN6C* | G | A |
| BMI | rs11847697 | *PRKD1* | T | C |
| BMI | rs12444979 | *GPRC5B* | C | T |
| BMI | rs13107325 | *SLC39A8* | T | C |
| BMI | rs1424233 | *MAF* | A | G |
| BMI | rs1514175 | *TNNI3K* | T | C |
| BMI | rs1555543 | *PTBP2* | C | A |
| BMI | rs17782313 | *MC4R* | C | T |
| BMI | rs1805081 | *NPC1* | A | G |
| BMI | rs206936 | *NUDT3* | G | A |
| BMI | rs2112347 | *FLJ35779* | T | G |
| BMI | rs2241423 | *MAP2K5* | G | A |
| BMI | rs2287019 | *QPCTL* | C | T |
| BMI | rs2568958 | *NEGR1* | A | G |
| BMI | rs2890652 | *LRP1B* | C | T |
| BMI | rs29941 | *KCTD15* | G | A |
| BMI | rs3810291 | *TMEM160* | A | G |
| BMI | rs4712652 | *LINC00340* | A | G |
| BMI | rs4771122 | *MTIF3* | G | A |
| BMI | rs4929949 | *RPL27A* | C | T |
| BMI | rs543874 | *SEC16B* | G | A |
| BMI | rs6013029 | *CTNNBL1* | T | G |
| BMI | rs6232 | *PCSK1* | G | A |
| BMI | rs6602024 | *PFKP* | A | G |
| BMI | rs713586 | *RBJ* | C | T |
| BMI | rs7647305 | *SFRS10* | C | T |
| BMI | rs9939609 | *FTO* | A | T |
| BMI/WC | rs10146997 | *NRXN3* | G | A |
| BMI/WC | rs1121980 | *FTO* | A | G |
| BMI/WC | rs7138803 | *FAIM2* | A | G |
| WC | rs12970134 | *MC4R* | A | G |
| WC | rs545854 | *MSRA* | G | C |
| WC | rs987237 | *TFAPB2* | G | A |
| WHR_BMI_ | rs1011731 | *DNM3-PIGC* | C | T |
| WHR_BMI_ | rs10195252 | *GRB14* | T | C |
| WHR_BMI_ | rs1055144 | *NFE2L3* | A | G |
| WHR_BMI_ | rs1294421 | *LY86* | G | T |
| WHR_BMI_ | rs1443512 | *HOXC13* | A | C |
| WHR_BMI_ | rs2605100 | *LYPLAL1* | G | A |
| WHR_BMI_ | rs4823006 | *ZNRF3-KREMEN1* | A | G |
| WHR_BMI_ | rs6784615 | *NISCH-STAB1* | T | C |
| WHR_BMI_ | rs6795735 | *ADAMTS9* | C | T |
| WHR_BMI_ | rs6861681 | *CPEB4* | A | G |
| WHR_BMI_ | rs6905288 | *VEGFA* | A | G |
| WHR_BMI_ | rs718314 | *ITPR2-SSPN* | C | T |
| WHR_BMI_ | rs9491696 | *RSPO3* | G | C |
| WHR_BMI_ | rs984222 | *TBX15-WARS2* | G | C |

Abbreviations: BMI, body mass index; WC, waist circumference; WHR_BMI_, Waist-hip ratio adjusted for BMI.

**Table S2A-J:** Interaction between 50 adiposity-related SNPs and dietary protein replacing either carbohydrate or fat in relation to change in BW or WC in the three cohorts. Corrected p-value: Adjusting for multiple testing through Bonferroni correction of the 50 test performed.

**Table S2A**

| Diet Cancer and Health cohort,  Change in BW (gram/y/5 % protein/risk allele). Protein replacing carbohydrate | | | | | | |
| --- | --- | --- | --- | --- | --- | --- |
| SNP | N | β | 95% CI | | p-value | corrected p-value |
| rs1011731 | 1817 | -69.8 | -168.6 | 28.9 | 0.166 | 1.00 |
| rs10146997 | 1872 | 111.5 | -7.1 | 230.1 | 0.065 | 1.00 |
| rs10195252 | 1857 | -39.0 | -133.8 | 55.8 | 0.420 | 1.00 |
| rs10508503 | 1879 | -47.1 | -226.2 | 131.9 | 0.606 | 1.00 |
| rs1055144 | 1876 | -30.2 | -149.9 | 89.5 | 0.621 | 1.00 |
| rs10838738 | 1867 | 4.5 | -95.1 | 104.1 | 0.929 | 1.00 |
| rs10913469 | 1875 | -121.1 | -239.2 | -2.9 | 0.045 | 1.00 |
| rs10938397 | 1872 | 37.0 | -61.0 | 135.1 | 0.459 | 1.00 |
| rs10968576 | 1868 | -47.7 | -151.5 | 56.1 | 0.368 | 1.00 |
| rs1121980 | 1881 | 49.1 | -44.1 | 142.2 | 0.302 | 1.00 |
| rs11847697 | 1881 | 283.9 | 36.2 | 531.6 | 0.025 | 1.00 |
| rs12444979 | 1873 | -44.8 | -183.2 | 93.5 | 0.525 | 1.00 |
| rs1294421 | 1885 | -14.3 | -110.5 | 81.9 | 0.771 | 1.00 |
| rs12970134 | 1865 | 21.5 | -83.0 | 125.9 | 0.687 | 1.00 |
| rs13107325 | 1870 | -30.6 | -263.2 | 202.0 | 0.796 | 1.00 |
| rs1424233 | 1878 | 78.4 | -17.2 | 173.9 | 0.108 | 1.00 |
| rs1443512 | 1887 | -34.3 | -143.5 | 75.0 | 0.539 | 1.00 |
| rs1514175 | 1872 | 100.3 | 5.2 | 195.4 | 0.039 | 1.00 |
| rs1555543 | 1874 | 79.1 | -11.7 | 169.9 | 0.088 | 1.00 |
| rs17782313 | 1881 | -2.8 | -111.0 | 105.5 | 0.960 | 1.00 |
| rs1805081 | 1874 | -63.0 | -156.1 | 30.0 | 0.184 | 1.00 |
| rs206936 | 1882 | -28.8 | -145.7 | 88.2 | 0.630 | 1.00 |
| rs2112347 | 1871 | 32.3 | -66.5 | 131.1 | 0.522 | 1.00 |
| rs2241423 | 1874 | -154.7 | -264.5 | -44.9 | 0.006 | 0.289 |
| rs2287019 | 1859 | -51.7 | -162.9 | 59.5 | 0.362 | 1.00 |
| rs2568958 | 1880 | -8.0 | -102.9 | 86.9 | 0.869 | 1.00 |
| rs2605100 | 1872 | 127.1 | 24.0 | 230.1 | 0.016 | 0.784 |
| rs2890652 | 1866 | -91.7 | -218.6 | 35.2 | 0.157 | 1.00 |
| rs29941 | 1872 | 10.4 | -84.0 | 104.7 | 0.829 | 1.00 |
| rs3810291 | 1879 | 97.3 | -1.6 | 196.2 | 0.054 | 1.00 |
| rs4712652 | 1851 | -11.7 | -104.9 | 81.5 | 0.806 | 1.00 |
| rs4771122 | 1849 | 46.9 | -58.7 | 152.5 | 0.384 | 1.00 |
| rs4823006 | 1875 | 64.6 | -30.6 | 159.7 | 0.183 | 1.00 |
| rs4836133 | 1874 | 86.2 | -1.7 | 174.0 | 0.055 | 1.00 |
| rs4923461 | 1882 | -26.1 | -138.9 | 86.6 | 0.650 | 1.00 |
| rs4929949 | 1850 | -22.5 | -115.1 | 70.1 | 0.634 | 1.00 |
| rs543874 | 1871 | -63.0 | -182.1 | 56.1 | 0.300 | 1.00 |
| rs545854 | 1873 | 5.1 | -118.2 | 128.4 | 0.936 | 1.00 |
| rs6013029 | 1874 | -41.5 | -253.8 | 170.9 | 0.702 | 1.00 |
| rs6232 | 1878 | -63.6 | -264.6 | 137.5 | 0.536 | 1.00 |
| rs6602024 | 1876 | 33.4 | -119.4 | 186.3 | 0.668 | 1.00 |
| rs6784615 | 1878 | -124.2 | -331.6 | 83.2 | 0.241 | 1.00 |
| rs6795735 | 1851 | 33.0 | -63.8 | 129.9 | 0.504 | 1.00 |
| rs6861681 | 1850 | -5.3 | -103.3 | 92.6 | 0.916 | 1.00 |
| rs6905288 | 1880 | -26.8 | -117.7 | 64.1 | 0.564 | 1.00 |
| rs713586 | 1875 | 54.4 | -39.1 | 147.9 | 0.254 | 1.00 |
| rs7138803 | 1874 | 2.6 | -91.9 | 97.0 | 0.957 | 1.00 |
| rs718314 | 1863 | 22.3 | -87.4 | 132.0 | 0.691 | 1.00 |
| rs7498665 | 1870 | 40.8 | -53.0 | 134.5 | 0.394 | 1.00 |
| rs7561317 | 1869 | 1.0 | -135.3 | 137.4 | 0.988 | 1.00 |
| rs7647305 | 1864 | 89.4 | -26.5 | 205.2 | 0.131 | 1.00 |
| rs780094 | 1869 | -20.4 | -128.2 | 87.4 | 0.711 | 1.00 |
| rs925946 | 1872 | -30.2 | -128.3 | 67.9 | 0.546 | 1.00 |
| rs9491696 | 1865 | 43.2 | -51.6 | 138.0 | 0.371 | 1.00 |
| rs984222 | 1880 | -16.3 | -114.9 | 82.3 | 0.746 | 1.00 |
| rs987237 | 1883 | 50.4 | -73.4 | 174.2 | 0.425 | 1.00 |
| rs9939609 | 2167 | 37.3 | -50.4 | 125.0 | 0.405 | 1.00 |

**Table S2B**

| Diet Cancer and Health cohort,  Change in BW (gram/y/5 E% protein/risk allele). Protein replacing fat | | | | | | |
| --- | --- | --- | --- | --- | --- | --- |
| SNP | N | β | 95% CI | | p-value | corrected p-value |
| rs1011731 | 1817 | -1.8 | -121.3 | 117.7 | 0.977 | 1.00 |
| rs10146997 | 1872 | 56.6 | -91.6 | 204.9 | 0.454 | 1.00 |
| rs10195252 | 1857 | -59.8 | -174.4 | 54.9 | 0.307 | 1.00 |
| rs10508503 | 1879 | 165.9 | -64.2 | 395.9 | 0.158 | 1.00 |
| rs1055144 | 1876 | -18.8 | -168.1 | 130.6 | 0.806 | 1.00 |
| rs10838738 | 1867 | -35.5 | -159.7 | 88.7 | 0.575 | 1.00 |
| rs10913469 | 1875 | -38.3 | -180.4 | 103.9 | 0.598 | 1.00 |
| rs10938397 | 1872 | -61.2 | -180.9 | 58.5 | 0.316 | 1.00 |
| rs10968576 | 1868 | 68.0 | -55.0 | 191.0 | 0.279 | 1.00 |
| rs1121980 | 1881 | -64.5 | -185.0 | 56.0 | 0.294 | 1.00 |
| rs11847697 | 1881 | 125.6 | -174.0 | 425.2 | 0.411 | 1.00 |
| rs12444979 | 1873 | -38.1 | -206.2 | 130.1 | 0.657 | 1.00 |
| rs1294421 | 1885 | -79.8 | -197.7 | 38.2 | 0.185 | 1.00 |
| rs12970134 | 1865 | -1.9 | -131.7 | 127.9 | 0.977 | 1.00 |
| rs13107325 | 1870 | -90.9 | -365.6 | 183.7 | 0.517 | 1.00 |
| rs1424233 | 1878 | -23.9 | -140.8 | 92.9 | 0.689 | 1.00 |
| rs1443512 | 1887 | 128.9 | -8.3 | 266.1 | 0.065 | 1.00 |
| rs1514175 | 1872 | -46.8 | -159.3 | 65.7 | 0.415 | 1.00 |
| rs1555543 | 1874 | 57.1 | -59.7 | 173.8 | 0.338 | 1.00 |
| rs17782313 | 1881 | -0.4 | -138.3 | 137.4 | 0.995 | 1.00 |
| rs1805081 | 1874 | 16.5 | -100.1 | 133.1 | 0.781 | 1.00 |
| rs206936 | 1882 | -34.9 | -180.4 | 110.7 | 0.639 | 1.00 |
| rs2112347 | 1871 | -13.8 | -135.2 | 107.6 | 0.824 | 1.00 |
| rs2241423 | 1874 | 15.3 | -119.0 | 149.5 | 0.826 | 1.00 |
| rs2287019 | 1859 | 116.2 | -29.7 | 262.0 | 0.119 | 1.00 |
| rs2568958 | 1880 | -46.1 | -163.8 | 71.6 | 0.443 | 1.00 |
| rs2605100 | 1872 | 5.2 | -119.9 | 130.4 | 0.935 | 1.00 |
| rs2890652 | 1866 | 59.3 | -95.5 | 214.1 | 0.453 | 1.00 |
| rs29941 | 1872 | -6.2 | -128.4 | 115.9 | 0.921 | 1.00 |
| rs3810291 | 1879 | -7.4 | -127.8 | 113.0 | 0.904 | 1.00 |
| rs4712652 | 1851 | -38.3 | -150.9 | 74.3 | 0.505 | 1.00 |
| rs4771122 | 1849 | 35.2 | -95.8 | 166.1 | 0.599 | 1.00 |
| rs4823006 | 1875 | -25.0 | -141.1 | 91.2 | 0.673 | 1.00 |
| rs4836133 | 1874 | 79.5 | -30.2 | 189.1 | 0.156 | 1.00 |
| rs4923461 | 1882 | -28.3 | -172.3 | 115.8 | 0.701 | 1.00 |
| rs4929949 | 1850 | 79.1 | -37.2 | 195.4 | 0.182 | 1.00 |
| rs543874 | 1871 | -84.6 | -227.8 | 58.6 | 0.247 | 1.00 |
| rs545854 | 1873 | 178.4 | 20.2 | 336.5 | 0.027 | 1.00 |
| rs6013029 | 1874 | -224.9 | -496.9 | 47.1 | 0.105 | 1.00 |
| rs6232 | 1878 | 67.6 | -134.6 | 269.7 | 0.513 | 1.00 |
| rs6602024 | 1876 | -328.2 | -510.9 | -145.5 | 0.001 | 0.022 |
| rs6784615 | 1878 | 285.6 | 25.5 | 545.7 | 0.031 | 1.00 |
| rs6795735 | 1851 | -160.6 | -285.3 | -36.0 | 0.012 | 0.578 |
| rs6861681 | 1850 | 67.3 | -56.4 | 190.9 | 0.287 | 1.00 |
| rs6905288 | 1880 | 40.9 | -75.6 | 157.3 | 0.491 | 1.00 |
| rs713586 | 1875 | -22.9 | -142.4 | 96.6 | 0.708 | 1.00 |
| rs7138803 | 1874 | -18.4 | -139.2 | 102.5 | 0.766 | 1.00 |
| rs718314 | 1863 | 13.1 | -122.0 | 148.2 | 0.850 | 1.00 |
| rs7498665 | 1870 | 37.4 | -78.5 | 153.2 | 0.527 | 1.00 |
| rs7561317 | 1869 | -61.7 | -224.5 | 101.1 | 0.458 | 1.00 |
| rs7647305 | 1864 | 78.9 | -70.9 | 228.6 | 0.302 | 1.00 |
| rs780094 | 1869 | -148.4 | -275.0 | -21.8 | 0.022 | 1.00 |
| rs925946 | 1872 | -26.7 | -149.4 | 95.9 | 0.670 | 1.00 |
| rs9491696 | 1865 | -74.8 | -188.5 | 38.9 | 0.198 | 1.00 |
| rs984222 | 1880 | -48.2 | -171.1 | 74.7 | 0.442 | 1.00 |
| rs987237 | 1883 | -127.9 | -281.6 | 25.9 | 0.103 | 1.00 |
| rs9939609 | 2167 | 15.9 | -96.3 | 128.0 | 0.782 | 1.00 |

**Table S2C**

| Diet Cancer and Health cohort,  Change in WC (mm/y/5 E% protein/risk allele). Protein replacing carbohydrate | | | | | | |
| --- | --- | --- | --- | --- | --- | --- |
| SNP | N | β | 95% CI | | p-value | corrected p-value |
| rs1011731 | 1783 | 0.3 | -0.9 | 1.5 | 0.631 | 1.00 |
| rs10146997 | 1838 | 0.7 | -0.7 | 2.1 | 0.336 | 1.00 |
| rs10195252 | 1822 | -0.4 | -1.5 | 0.7 | 0.466 | 1.00 |
| rs10508503 | 1845 | 0.9 | -1.2 | 3.0 | 0.400 | 1.00 |
| rs1055144 | 1842 | -1.0 | -2.4 | 0.4 | 0.167 | 1.00 |
| rs10838738 | 1832 | -0.6 | -1.8 | 0.5 | 0.284 | 1.00 |
| rs10913469 | 1840 | -0.2 | -1.6 | 1.2 | 0.809 | 1.00 |
| rs10938397 | 1838 | 0.8 | -0.3 | 2.0 | 0.153 | 1.00 |
| rs10968576 | 1833 | -0.6 | -1.8 | 0.6 | 0.330 | 1.00 |
| rs1121980 | 1847 | -0.3 | -1.4 | 0.8 | 0.576 | 1.00 |
| rs11847697 | 1846 | 0.3 | -2.7 | 3.2 | 0.852 | 1.00 |
| rs12444979 | 1838 | 0.7 | -0.9 | 2.4 | 0.388 | 1.00 |
| rs1294421 | 1850 | -0.4 | -1.6 | 0.7 | 0.444 | 1.00 |
| rs12970134 | 1830 | -0.2 | -1.5 | 1.0 | 0.733 | 1.00 |
| rs13107325 | 1835 | 0.8 | -2.0 | 3.6 | 0.580 | 1.00 |
| rs1424233 | 1843 | 0.8 | -0.3 | 1.9 | 0.164 | 1.00 |
| rs1443512 | 1852 | 1.5 | 0.2 | 2.8 | 0.023 | 1.00 |
| rs1514175 | 1839 | 0.6 | -0.5 | 1.8 | 0.273 | 1.00 |
| rs1555543 | 1839 | 1.0 | -0.1 | 2.1 | 0.070 | 1.00 |
| rs17782313 | 1846 | -0.3 | -1.6 | 0.9 | 0.606 | 1.00 |
| rs1805081 | 1839 | -0.3 | -1.4 | 0.8 | 0.637 | 1.00 |
| rs206936 | 1847 | -0.1 | -1.5 | 1.3 | 0.914 | 1.00 |
| rs2112347 | 1836 | 0.6 | -0.6 | 1.8 | 0.315 | 1.00 |
| rs2241423 | 1839 | -1.6 | -2.9 | -0.3 | 0.014 | 0.722 |
| rs2287019 | 1824 | 0.8 | -0.5 | 2.2 | 0.206 | 1.00 |
| rs2568958 | 1845 | 0.3 | -0.8 | 1.4 | 0.596 | 1.00 |
| rs2605100 | 1837 | 1.1 | -0.2 | 2.3 | 0.091 | 1.00 |
| rs2890652 | 1831 | -1.0 | -2.5 | 0.5 | 0.202 | 1.00 |
| rs29941 | 1837 | 0.4 | -0.7 | 1.6 | 0.452 | 1.00 |
| rs3810291 | 1844 | -0.1 | -1.3 | 1.1 | 0.871 | 1.00 |
| rs4712652 | 1817 | 0.4 | -0.7 | 1.5 | 0.493 | 1.00 |
| rs4771122 | 1814 | -1.1 | -2.3 | 0.2 | 0.094 | 1.00 |
| rs4823006 | 1840 | 0.1 | -1.0 | 1.3 | 0.821 | 1.00 |
| rs4836133 | 1840 | -0.8 | -1.9 | 0.2 | 0.121 | 1.00 |
| rs4923461 | 1848 | 0.1 | -1.3 | 1.4 | 0.901 | 1.00 |
| rs4929949 | 1816 | -0.4 | -1.5 | 0.7 | 0.447 | 1.00 |
| rs543874 | 1837 | -0.7 | -2.1 | 0.7 | 0.352 | 1.00 |
| rs545854 | 1838 | -0.4 | -1.9 | 1.0 | 0.576 | 1.00 |
| rs6013029 | 1839 | 0.1 | -2.5 | 2.6 | 0.965 | 1.00 |
| rs6232 | 1844 | -0.4 | -2.7 | 2.0 | 0.763 | 1.00 |
| rs6602024 | 1841 | 2.0 | 0.2 | 3.8 | 0.033 | 1.00 |
| rs6784615 | 1843 | 2.0 | -0.4 | 4.5 | 0.107 | 1.00 |
| rs6795735 | 1817 | -0.7 | -1.8 | 0.5 | 0.242 | 1.00 |
| rs6861681 | 1816 | 0.3 | -0.9 | 1.5 | 0.630 | 1.00 |
| rs6905288 | 1845 | 0.3 | -0.8 | 1.4 | 0.568 | 1.00 |
| rs713586 | 1840 | -0.2 | -1.3 | 1.0 | 0.780 | 1.00 |
| rs7138803 | 1840 | -1.2 | -2.4 | -0.1 | 0.030 | 1.00 |
| rs718314 | 1828 | -0.3 | -1.6 | 1.0 | 0.632 | 1.00 |
| rs7498665 | 1835 | -0.8 | -1.9 | 0.3 | 0.164 | 1.00 |
| rs7561317 | 1834 | 2.4 | 0.8 | 4.0 | 0.004 | 0.181 |
| rs7647305 | 1831 | 0.2 | -1.2 | 1.6 | 0.768 | 1.00 |
| rs780094 | 1834 | -0.8 | -2.1 | 0.5 | 0.230 | 1.00 |
| rs925946 | 1837 | 1.0 | -0.2 | 2.1 | 0.101 | 1.00 |
| rs9491696 | 1830 | 1.0 | -0.1 | 2.1 | 0.083 | 1.00 |
| rs984222 | 1845 | 0.4 | -0.8 | 1.6 | 0.487 | 1.00 |
| rs987237 | 1850 | -0.3 | -1.8 | 1.2 | 0.699 | 1.00 |
| rs9939609 | 2128 | -0.5 | -1.6 | 0.5 | 0.327 | 1.00 |

**Table S2D**

| Diet Cancer and Health cohort,  Change in WC (mm/y/5 E% protein/risk allele). Protein replacing fat | | | | | | |
| --- | --- | --- | --- | --- | --- | --- |
| SNP | N | β | 95% CI | | p-value | corrected p-value |
| rs1011731 | 1783 | -0.7 | -2.1 | 0.8 | 0.358 | 1.00 |
| rs10146997 | 1838 | -0.2 | -1.9 | 1.6 | 0.860 | 1.00 |
| rs10195252 | 1822 | 0.6 | -0.8 | 2.0 | 0.397 | 1.00 |
| rs10508503 | 1845 | -3.9 | -6.6 | -1.1 | 0.006 | 0.308 |
| rs1055144 | 1842 | -0.6 | -2.4 | 1.2 | 0.505 | 1.00 |
| rs10838738 | 1832 | 0.9 | -0.6 | 2.4 | 0.238 | 1.00 |
| rs10913469 | 1840 | 1.0 | -0.7 | 2.7 | 0.231 | 1.00 |
| rs10938397 | 1838 | -0.1 | -1.5 | 1.3 | 0.907 | 1.00 |
| rs10968576 | 1833 | 0.4 | -1.0 | 1.9 | 0.571 | 1.00 |
| rs1121980 | 1847 | -0.8 | -2.2 | 0.6 | 0.264 | 1.00 |
| rs11847697 | 1846 | 3.0 | -0.6 | 6.5 | 0.105 | 1.00 |
| rs12444979 | 1838 | 0.1 | -1.9 | 2.1 | 0.944 | 1.00 |
| rs1294421 | 1850 | 0.7 | -0.7 | 2.1 | 0.316 | 1.00 |
| rs12970134 | 1830 | 0.1 | -1.5 | 1.6 | 0.926 | 1.00 |
| rs13107325 | 1835 | 1.6 | -1.6 | 4.9 | 0.332 | 1.00 |
| rs1424233 | 1843 | -0.4 | -1.8 | 1.0 | 0.595 | 1.00 |
| rs1443512 | 1852 | -1.5 | -3.1 | 0.2 | 0.075 | 1.00 |
| rs1514175 | 1839 | -0.3 | -1.6 | 1.1 | 0.677 | 1.00 |
| rs1555543 | 1839 | -0.2 | -1.6 | 1.2 | 0.793 | 1.00 |
| rs17782313 | 1846 | 0.0 | -1.7 | 1.6 | 0.979 | 1.00 |
| rs1805081 | 1839 | -0.5 | -1.9 | 0.9 | 0.467 | 1.00 |
| rs206936 | 1847 | -0.3 | -2.1 | 1.4 | 0.694 | 1.00 |
| rs2112347 | 1836 | -0.4 | -1.9 | 1.0 | 0.567 | 1.00 |
| rs2241423 | 1839 | 0.9 | -0.7 | 2.5 | 0.260 | 1.00 |
| rs2287019 | 1824 | -0.3 | -2.1 | 1.4 | 0.729 | 1.00 |
| rs2568958 | 1845 | 0.4 | -1.0 | 1.8 | 0.584 | 1.00 |
| rs2605100 | 1837 | -0.5 | -1.9 | 1.0 | 0.552 | 1.00 |
| rs2890652 | 1831 | -1.3 | -3.1 | 0.6 | 0.181 | 1.00 |
| rs29941 | 1837 | -0.4 | -1.8 | 1.1 | 0.634 | 1.00 |
| rs3810291 | 1844 | -0.7 | -2.1 | 0.8 | 0.366 | 1.00 |
| rs4712652 | 1817 | 0.7 | -0.6 | 2.0 | 0.293 | 1.00 |
| rs4771122 | 1814 | 0.9 | -0.6 | 2.5 | 0.234 | 1.00 |
| rs4823006 | 1840 | 0.8 | -0.6 | 2.1 | 0.282 | 1.00 |
| rs4836133 | 1840 | -0.3 | -1.6 | 1.0 | 0.607 | 1.00 |
| rs4923461 | 1848 | -0.9 | -2.7 | 0.8 | 0.284 | 1.00 |
| rs4929949 | 1816 | 0.5 | -0.9 | 1.9 | 0.458 | 1.00 |
| rs543874 | 1837 | 0.4 | -1.3 | 2.1 | 0.681 | 1.00 |
| rs545854 | 1838 | -0.1 | -2.0 | 1.8 | 0.897 | 1.00 |
| rs6013029 | 1839 | 0.7 | -2.6 | 3.9 | 0.694 | 1.00 |
| rs6232 | 1844 | -1.7 | -4.1 | 0.7 | 0.157 | 1.00 |
| rs6602024 | 1841 | -0.7 | -2.9 | 1.5 | 0.534 | 1.00 |
| rs6784615 | 1843 | -2.0 | -5.1 | 1.1 | 0.204 | 1.00 |
| rs6795735 | 1817 | 0.2 | -1.2 | 1.7 | 0.742 | 1.00 |
| rs6861681 | 1816 | -0.2 | -1.7 | 1.3 | 0.778 | 1.00 |
| rs6905288 | 1845 | -1.8 | -3.2 | -0.5 | 0.009 | 0.454 |
| rs713586 | 1840 | 0.2 | -1.2 | 1.6 | 0.789 | 1.00 |
| rs7138803 | 1840 | 0.5 | -1.0 | 1.9 | 0.532 | 1.00 |
| rs718314 | 1828 | 0.4 | -1.2 | 2.0 | 0.646 | 1.00 |
| rs7498665 | 1835 | -1.0 | -2.3 | 0.4 | 0.173 | 1.00 |
| rs7561317 | 1834 | 0.6 | -1.3 | 2.5 | 0.545 | 1.00 |
| rs7647305 | 1831 | 0.1 | -1.7 | 1.8 | 0.947 | 1.00 |
| rs780094 | 1834 | 1.5 | -0.1 | 3.0 | 0.059 | 1.00 |
| rs925946 | 1837 | -0.1 | -1.6 | 1.3 | 0.859 | 1.00 |
| rs9491696 | 1830 | 1.2 | -0.1 | 2.6 | 0.075 | 1.00 |
| rs984222 | 1845 | -0.4 | -1.8 | 1.1 | 0.600 | 1.00 |
| rs987237 | 1850 | -1.0 | -2.9 | 0.8 | 0.264 | 1.00 |
| rs9939609 | 2128 | -0.8 | -2.2 | 0.5 | 0.224 | 1.00 |

**Table S2E**

| INTER99 cohort,  Change in BW (gram/y/5 E% protein/risk allele). Protein replacing carbohydrate | | | | | | |
| --- | --- | --- | --- | --- | --- | --- |
| SNP | N | β | 95% CI | | p-value | corrected p-value |
| rs1011731 | 3484 | -33.7985 | -87.23 | 19.633 | 0.215 | 1.00 |
| rs10146997 | 3520 | 31.5775 | -36.602 | 99.757 | 0.364 | 1.00 |
| rs10195252 | 3484 | -16.092 | -69.5615 | 37.377 | 0.555 | 1.00 |
| rs10508503 | 3550 | -53.918 | -150.633 | 42.797 | 0.275 | 1.00 |
| rs1055144 | 3498 | -4.345 | -75.2505 | 66.561 | 0.904 | 1.00 |
| rs10838738 | 3520 | -9.027 | -64.5385 | 46.485 | 0.750 | 1.00 |
| rs10938397 | 3497 | 25.5375 | -28.2875 | 79.3625 | 0.352 | 1.00 |
| rs10968576 | 3480 | 59.5705 | -0.3695 | 119.5105 | 0.051 | 1.00 |
| rs1121980 | 3227 | 41.354 | -15.732 | 98.44 | 0.156 | 1.00 |
| rs11847697 | 3511 | 63.0035 | -71.7555 | 197.7625 | 0.360 | 1.00 |
| rs12444979 | 3508 | -29.3595 | -107.455 | 48.7355 | 0.461 | 1.00 |
| rs1294421 | 3479 | 33.8245 | -21.7075 | 89.3565 | 0.233 | 1.00 |
| rs12970134 | 3515 | -25.738 | -85.869 | 34.393 | 0.402 | 1.00 |
| rs13107325 | 3505 | 70.3675 | -50.79 | 191.5255 | 0.255 | 1.00 |
| rs1424233 | 3517 | 45.982 | -6.6755 | 98.6395 | 0.087 | 1.00 |
| rs1443512 | 3472 | -1.486 | -66.2675 | 63.2955 | 0.964 | 1.00 |
| rs1514175 | 3511 | 11.097 | -41.8565 | 64.0505 | 0.681 | 1.00 |
| rs1555543 | 3486 | 15.0825 | -36.52 | 66.685 | 0.567 | 1.00 |
| rs17782313 | 3533 | 12.0475 | -50.562 | 74.6565 | 0.706 | 1.00 |
| rs1805081 | 3542 | -35.849 | -89.237 | 17.539 | 0.188 | 1.00 |
| rs206936 | 3470 | -61.9525 | -129.194 | 5.2885 | 0.071 | 1.00 |
| rs2112347 | 3523 | 11.346 | -42.8165 | 65.508 | 0.681 | 1.00 |
| rs2241423 | 3493 | 50.691 | -12.99 | 114.372 | 0.119 | 1.00 |
| rs2287019 | 3455 | 4.159 | -63.708 | 72.026 | 0.904 | 1.00 |
| rs2568958 | 3226 | -38.0625 | -95.01 | 18.885 | 0.190 | 1.00 |
| rs2605100 | 3512 | -47.8405 | -107.273 | 11.5915 | 0.115 | 1.00 |
| rs2890652 | 3514 | -20.7985 | -93.1385 | 51.5415 | 0.573 | 1.00 |
| rs29941 | 3229 | -97.1225 | -158.643 | -35.6025 | 0.002 | 0.099 |
| rs3810291 | 3466 | 10.337 | -47.2285 | 67.9025 | 0.725 | 1.00 |
| rs4712652 | 3513 | -51.071 | -103.315 | 1.1725 | 0.055 | 1.00 |
| rs4771122 | 3483 | 23.3715 | -36.3535 | 83.097 | 0.443 | 1.00 |
| rs4823006 | 3475 | 33.1095 | -21.429 | 87.6485 | 0.234 | 1.00 |
| rs4929949 | 3511 | 66.001 | 12.161 | 119.8415 | 0.016 | 0.814 |
| rs543874 | 3229 | 0.896 | -66.74 | 68.5315 | 0.979 | 1.00 |
| rs545854 | 3534 | -52.9825 | -122.339 | 16.3735 | 0.134 | 1.00 |
| rs6013029 | 3543 | 121.6025 | -6.132 | 249.337 | 0.062 | 1.00 |
| rs6232 | 3520 | -42.6825 | -150.813 | 65.447 | 0.439 | 1.00 |
| rs6602024 | 3514 | -65.4365 | -152.614 | 21.7415 | 0.141 | 1.00 |
| rs6784615 | 3515 | -16.615 | -141.242 | 108.012 | 0.794 | 1.00 |
| rs6795735 | 3506 | -18.707 | -71.24 | 33.826 | 0.485 | 1.00 |
| rs6861681 | 3501 | -23.2935 | -82.4775 | 35.89 | 0.441 | 1.00 |
| rs6905288 | 3494 | 56.3495 | 3.7095 | 108.99 | 0.036 | 1.00 |
| rs713586 | 3476 | -44.3145 | -97.8165 | 9.187 | 0.105 | 1.00 |
| rs7138803 | 3227 | -29.334 | -87.127 | 28.4595 | 0.320 | 1.00 |
| rs718314 | 3505 | -43.828 | -105.954 | 18.2975 | 0.167 | 1.00 |
| rs7647305 | 3229 | 8.8495 | -64.803 | 82.502 | 0.814 | 1.00 |
| rs9491696 | 3445 | -7.035 | -58.866 | 44.7965 | 0.790 | 1.00 |
| rs984222 | 3493 | -56.459 | -111.655 | -1.2635 | 0.045 | 1.00 |
| rs987237 | 3506 | -42.12 | -113.855 | 29.615 | 0.250 | 1.00 |
| rs9939609 | 3478 | 39.792 | -14.622 | 94.2065 | 0.152 | 1.00 |

**Table S2F**

| INTER99 cohort,  Change in BW (gram/y/5 E% protein/risk allele). Protein replacing fat | | | | | | |
| --- | --- | --- | --- | --- | --- | --- |
| SNP | N | β | 95% CI | | p-value | corrected p-value |
| rs1011731 | 3484 | 22.566 | -38.321 | 83.4535 | 0.468 | 1.00 |
| rs10146997 | 3520 | 63.5795 | -14.6995 | 141.858 | 0.111 | 1.00 |
| rs10195252 | 3484 | 36.278 | -25.4525 | 98.0085 | 0.249 | 1.00 |
| rs10508503 | 3550 | 73.5925 | -37.86 | 185.0445 | 0.196 | 1.00 |
| rs1055144 | 3498 | -18.746 | -99.054 | 61.562 | 0.647 | 1.00 |
| rs10838738 | 3520 | 7.489 | -55.223 | 70.201 | 0.815 | 1.00 |
| rs10938397 | 3497 | -7.263 | -68.145 | 53.6185 | 0.815 | 1.00 |
| rs10968576 | 3480 | -27.9855 | -92.2405 | 36.27 | 0.393 | 1.00 |
| rs1121980 | 3227 | -64.1795 | -129.4 | 1.0415 | 0.054 | 1.00 |
| rs11847697 | 3511 | -52.255 | -207.305 | 102.7955 | 0.509 | 1.00 |
| rs12444979 | 3508 | -3.689 | -93.7615 | 86.383 | 0.936 | 1.00 |
| rs1294421 | 3479 | -30.099 | -93.129 | 32.931 | 0.349 | 1.00 |
| rs12970134 | 3515 | 17.597 | -51.24 | 86.4345 | 0.616 | 1.00 |
| rs13107325 | 3505 | -35.7925 | -179.583 | 107.9975 | 0.626 | 1.00 |
| rs1424233 | 3517 | -60.644 | -120.986 | -0.3015 | 0.049 | 1.00 |
| rs1443512 | 3472 | 55.9595 | -16.469 | 128.388 | 0.130 | 1.00 |
| rs1514175 | 3511 | -11.277 | -70.4175 | 47.864 | 0.709 | 1.00 |
| rs1555543 | 3486 | -61.281 | -120.75 | -1.812 | 0.043 | 1.00 |
| rs17782313 | 3533 | 0.4855 | -70.439 | 71.41 | 0.989 | 1.00 |
| rs1805081 | 3542 | -17.49 | -78.8515 | 43.8715 | 0.576 | 1.00 |
| rs206936 | 3470 | 18.8915 | -60.2915 | 98.074 | 0.640 | 1.00 |
| rs2112347 | 3523 | 21.842 | -39.663 | 83.347 | 0.486 | 1.00 |
| rs2241423 | 3493 | -38.468 | -111.938 | 35.0015 | 0.305 | 1.00 |
| rs2287019 | 3455 | 30.1375 | -47.218 | 107.4935 | 0.445 | 1.00 |
| rs2568958 | 3226 | 24.8195 | -39.3125 | 88.9515 | 0.448 | 1.00 |
| rs2605100 | 3512 | 71.952 | 4.385 | 139.519 | 0.037 | 1.00 |
| rs2890652 | 3514 | 13.3635 | -66.0145 | 92.742 | 0.741 | 1.00 |
| rs29941 | 3229 | 8.7765 | -59.6225 | 77.176 | 0.801 | 1.00 |
| rs3810291 | 3466 | 18.0945 | -46.8705 | 83.0595 | 0.585 | 1.00 |
| rs4712652 | 3513 | 75.885 | 16.4055 | 135.3645 | 0.012 | 0.620 |
| rs4771122 | 3483 | -32.237 | -99.146 | 34.6715 | 0.345 | 1.00 |
| rs4823006 | 3475 | -5.497 | -65.5625 | 54.569 | 0.858 | 1.00 |
| rs4929949 | 3511 | -10.4035 | -70.5225 | 49.7155 | 0.735 | 1.00 |
| rs543874 | 3229 | 103.6835 | 26.4345 | 180.933 | 0.009 | 0.426 |
| rs545854 | 3534 | 21.0795 | -59.03 | 101.1885 | 0.606 | 1.00 |
| rs6013029 | 3543 | 39.579 | -101.52 | 180.6775 | 0.583 | 1.00 |
| rs6232 | 3520 | 20.577 | -103.04 | 144.194 | 0.744 | 1.00 |
| rs6602024 | 3514 | 60.893 | -34.8845 | 156.671 | 0.213 | 1.00 |
| rs6784615 | 3515 | 92.422 | -51.059 | 235.903 | 0.207 | 1.00 |
| rs6795735 | 3506 | 15.094 | -45.5155 | 75.704 | 0.626 | 1.00 |
| rs6861681 | 3501 | 15.9705 | -49.641 | 81.582 | 0.633 | 1.00 |
| rs6905288 | 3494 | -18.9285 | -79.698 | 41.841 | 0.542 | 1.00 |
| rs713586 | 3476 | -29.9265 | -89.358 | 29.505 | 0.324 | 1.00 |
| rs7138803 | 3227 | 4.1085 | -60.646 | 68.863 | 0.901 | 1.00 |
| rs718314 | 3505 | 13.995 | -55.6385 | 83.629 | 0.694 | 1.00 |
| rs7647305 | 3229 | 5.698 | -75.89 | 87.286 | 0.891 | 1.00 |
| rs9491696 | 3445 | 8.3375 | -51.5695 | 68.244 | 0.785 | 1.00 |
| rs984222 | 3493 | -12.735 | -76.058 | 50.588 | 0.694 | 1.00 |
| rs987237 | 3506 | 69.5405 | -9.488 | 148.5685 | 0.085 | 1.00 |
| rs9939609 | 3478 | -58.516 | -120.914 | 3.881 | 0.066 | 1.00 |

**Table S2G**

| INTER99 cohort,  Change in WC (mm/year/5 E% protein/risk allele). Protein replacing carbohydrate | | | | | | |
| --- | --- | --- | --- | --- | --- | --- |
| SNP | N | β | 95% CI | | p-value | corrected p-value |
| rs1011731 | 3029 | -48.0465 | -96.511 | 0.418 | 0.052 | 1.00 |
| rs10146997 | 3061 | 21.389 | -39.689 | 82.4675 | 0.493 | 1.00 |
| rs10195252 | 3031 | -4.0065 | -52.182 | 44.1685 | 0.871 | 1.00 |
| rs10508503 | 3088 | -34.2845 | -120.03 | 51.4615 | 0.433 | 1.00 |
| rs1055144 | 3039 | -12.0245 | -74.404 | 50.3555 | 0.706 | 1.00 |
| rs10838738 | 3061 | -8.812 | -57.6495 | 40.0255 | 0.724 | 1.00 |
| rs10938397 | 3044 | 44.8125 | -3.308 | 92.9335 | 0.068 | 1.00 |
| rs10968576 | 3028 | 14.098 | -39.4105 | 67.6065 | 0.606 | 1.00 |
| rs1121980 | 2794 | -7.4925 | -58.3695 | 43.385 | 0.773 | 1.00 |
| rs11847697 | 3055 | 14.303 | -111.523 | 140.1295 | 0.824 | 1.00 |
| rs12444979 | 3055 | 57.1405 | -12.446 | 126.7265 | 0.108 | 1.00 |
| rs1294421 | 3024 | -47.9235 | -97.709 | 1.8615 | 0.059 | 1.00 |
| rs12970134 | 3057 | 9.5655 | -43.7405 | 62.8715 | 0.725 | 1.00 |
| rs13107325 | 3049 | -32.09 | -143.797 | 79.617 | 0.573 | 1.00 |
| rs1424233 | 3056 | -2.892 | -50.376 | 44.592 | 0.905 | 1.00 |
| rs1443512 | 3022 | -1.358 | -58.879 | 56.163 | 0.963 | 1.00 |
| rs1514175 | 3055 | -10.239 | -58.6125 | 38.1345 | 0.678 | 1.00 |
| rs1555543 | 3029 | -10.6255 | -56.029 | 34.7785 | 0.647 | 1.00 |
| rs17782313 | 3075 | 4.338 | -52.059 | 60.735 | 0.880 | 1.00 |
| rs1805081 | 3080 | 6.63 | -41.069 | 54.329 | 0.785 | 1.00 |
| rs206936 | 3016 | 5.6025 | -54.7095 | 65.9145 | 0.856 | 1.00 |
| rs2112347 | 3063 | -0.5415 | -49.4035 | 48.3205 | 0.983 | 1.00 |
| rs2241423 | 3042 | 37.83 | -19.387 | 95.047 | 0.195 | 1.00 |
| rs2287019 | 3007 | -3.702 | -64.478 | 57.074 | 0.905 | 1.00 |
| rs2568958 | 2794 | -5.4105 | -55.647 | 44.8255 | 0.833 | 1.00 |
| rs2605100 | 3058 | -8.1865 | -62.329 | 45.9555 | 0.767 | 1.00 |
| rs2890652 | 3056 | -34.059 | -99.1655 | 31.0475 | 0.305 | 1.00 |
| rs29941 | 2796 | -12.3205 | -66.975 | 42.334 | 0.659 | 1.00 |
| rs3810291 | 3014 | -17.141 | -68.3905 | 34.108 | 0.512 | 1.00 |
| rs4712652 | 3050 | -20.1285 | -67.398 | 27.141 | 0.404 | 1.00 |
| rs4771122 | 3035 | 0.0935 | -52.925 | 53.1115 | 0.997 | 1.00 |
| rs4823006 | 3020 | -5.3225 | -54.792 | 44.147 | 0.833 | 1.00 |
| rs4929949 | 3054 | -23.2905 | -71.205 | 24.624 | 0.341 | 1.00 |
| rs543874 | 2796 | 15.711 | -44.551 | 75.973 | 0.609 | 1.00 |
| rs545854 | 3077 | 26.146 | -37.4805 | 89.773 | 0.421 | 1.00 |
| rs6013029 | 3086 | -50.546 | -161.285 | 60.1925 | 0.371 | 1.00 |
| rs6232 | 3064 | -32.4085 | -128.572 | 63.7545 | 0.509 | 1.00 |
| rs6602024 | 3063 | 73.905 | -3.4725 | 151.282 | 0.061 | 1.00 |
| rs6784615 | 3057 | 42.3995 | -71.398 | 156.1975 | 0.465 | 1.00 |
| rs6795735 | 3053 | -21.437 | -68.416 | 25.5425 | 0.371 | 1.00 |
| rs6861681 | 3043 | 38.0835 | -14.57 | 90.7375 | 0.156 | 1.00 |
| rs6905288 | 3041 | 36.496 | -11.0225 | 84.0145 | 0.132 | 1.00 |
| rs713586 | 3021 | 33.933 | -14.9945 | 82.86 | 0.174 | 1.00 |
| rs7138803 | 2795 | 47.355 | -3.6895 | 98.399 | 0.069 | 1.00 |
| rs718314 | 3048 | -25.5515 | -80.694 | 29.5915 | 0.364 | 1.00 |
| rs7647305 | 2796 | -21.481 | -87.0485 | 44.087 | 0.521 | 1.00 |
| rs9491696 | 3001 | -17.2825 | -63.686 | 29.121 | 0.465 | 1.00 |
| rs984222 | 3035 | 18.7185 | -30.647 | 68.0845 | 0.457 | 1.00 |
| rs987237 | 3054 | 13.992 | -50.9975 | 78.9815 | 0.673 | 1.00 |
| rs9939609 | 3023 | -4.099 | -53.311 | 45.1125 | 0.870 | 1.00 |

**Table S2H**

| INTER99 cohort,  Change in WC (mm/y/5 E% protein/risk allele). Protein replacing fat | | | | | | |
| --- | --- | --- | --- | --- | --- | --- |
| SNP | N | β | 95% CI | | p-value | corrected p-value |
| rs1011731 | 3029 | 42.3425 | -13.054 | 97.739 | 0.134 | 1.00 |
| rs10146997 | 3061 | 60.939 | -9.2865 | 131.165 | 0.089 | 1.00 |
| rs10195252 | 3031 | 6.7525 | -48.5125 | 62.0175 | 0.811 | 1.00 |
| rs10508503 | 3088 | 2.018 | -98.9825 | 103.019 | 0.969 | 1.00 |
| rs1055144 | 3039 | -24.4935 | -94.8375 | 45.8505 | 0.495 | 1.00 |
| rs10838738 | 3061 | 52.927 | -2.4535 | 108.3075 | 0.061 | 1.00 |
| rs10938397 | 3044 | -55.2735 | -109.492 | -1.0555 | 0.046 | 1.00 |
| rs10968576 | 3028 | 8.841 | -48.628 | 66.31 | 0.763 | 1.00 |
| rs1121980 | 2794 | -3.0365 | -60.941 | 54.8675 | 0.918 | 1.00 |
| rs11847697 | 3055 | -51.9105 | -189.15 | 85.329 | 0.459 | 1.00 |
| rs12444979 | 3055 | -5.4285 | -84.285 | 73.428 | 0.893 | 1.00 |
| rs1294421 | 3024 | 37.634 | -19.094 | 94.3615 | 0.194 | 1.00 |
| rs12970134 | 3057 | 12.5285 | -48.3575 | 73.414 | 0.687 | 1.00 |
| rs13107325 | 3049 | 15.247 | -124.606 | 155.0995 | 0.831 | 1.00 |
| rs1424233 | 3056 | 64.0965 | 10.643 | 117.5495 | 0.019 | 0.938 |
| rs1443512 | 3022 | 26.257 | -38.08 | 90.594 | 0.424 | 1.00 |
| rs1514175 | 3055 | -35.711 | -88.2095 | 16.788 | 0.183 | 1.00 |
| rs1555543 | 3029 | 34.2735 | -19.24 | 87.787 | 0.209 | 1.00 |
| rs17782313 | 3075 | 4.1755 | -59.392 | 67.743 | 0.898 | 1.00 |
| rs1805081 | 3080 | -12.3205 | -66.731 | 42.0895 | 0.657 | 1.00 |
| rs206936 | 3016 | 0.513 | -70.5335 | 71.559 | 0.989 | 1.00 |
| rs2112347 | 3063 | -13.7385 | -68.6095 | 41.132 | 0.624 | 1.00 |
| rs2241423 | 3042 | 13.893 | -51.564 | 79.3495 | 0.677 | 1.00 |
| rs2287019 | 3007 | -0.286 | -69.221 | 68.649 | 0.994 | 1.00 |
| rs2568958 | 2794 | 25.9235 | -30.435 | 82.282 | 0.367 | 1.00 |
| rs2605100 | 3058 | 16.8395 | -43.75 | 77.429 | 0.586 | 1.00 |
| rs2890652 | 3056 | 49.811 | -21.3725 | 120.9945 | 0.170 | 1.00 |
| rs29941 | 2796 | 27.371 | -32.802 | 87.5435 | 0.373 | 1.00 |
| rs3810291 | 3014 | 23.9045 | -34.2785 | 82.0875 | 0.421 | 1.00 |
| rs4712652 | 3050 | 15.743 | -37.5415 | 69.0275 | 0.563 | 1.00 |
| rs4771122 | 3035 | -27.6275 | -87.3005 | 32.046 | 0.364 | 1.00 |
| rs4823006 | 3020 | -37.288 | -91.245 | 16.669 | 0.176 | 1.00 |
| rs4929949 | 3054 | 32.2475 | -21.238 | 85.733 | 0.237 | 1.00 |
| rs543874 | 2796 | 25.282 | -45.3585 | 95.9225 | 0.483 | 1.00 |
| rs545854 | 3077 | 22.316 | -49.7385 | 94.3705 | 0.544 | 1.00 |
| rs6013029 | 3086 | 80.027 | -42.028 | 202.0825 | 0.199 | 1.00 |
| rs6232 | 3064 | 50.718 | -59.3205 | 160.7565 | 0.366 | 1.00 |
| rs6602024 | 3063 | 21.264 | -64.6575 | 107.186 | 0.628 | 1.00 |
| rs6784615 | 3057 | -51.932 | -180.014 | 76.1495 | 0.427 | 1.00 |
| rs6795735 | 3053 | 7.9705 | -46.12 | 62.061 | 0.773 | 1.00 |
| rs6861681 | 3043 | -32.4785 | -91.0035 | 26.046 | 0.277 | 1.00 |
| rs6905288 | 3041 | -38.319 | -92.732 | 16.0935 | 0.168 | 1.00 |
| rs713586 | 3021 | -75.132 | -128.763 | -21.501 | 0.006 | 0.302 |
| rs7138803 | 2795 | -45.039 | -102.468 | 12.3895 | 0.124 | 1.00 |
| rs718314 | 3048 | 2.6275 | -59.019 | 64.2735 | 0.933 | 1.00 |
| rs7647305 | 2796 | -56.975 | -128.662 | 14.7115 | 0.119 | 1.00 |
| rs9491696 | 3001 | 2.84 | -50.5515 | 56.231 | 0.917 | 1.00 |
| rs984222 | 3035 | -36.6765 | -93.17 | 19.817 | 0.203 | 1.00 |
| rs987237 | 3054 | 2.826 | -68.308 | 73.9595 | 0.938 | 1.00 |
| rs9939609 | 3023 | -12.0055 | -68.328 | 44.317 | 0.676 | 1.00 |

**Table S2I**

| MONICA cohort,  Change in BW (gram/y/5 E% protein/risk allele). Protein replacing carbohydrate | | | | | | |
| --- | --- | --- | --- | --- | --- | --- |
| SNP | N | β | 95% CI | | p-value | corrected p-value |
| rs1011731 | 1245 | 80.928 | -16.7185 | 178.575 | 0.104 | 1.00 |
| rs10146997 | 1252 | 103.659 | -7.2755 | 214.5935 | 0.067 | 1.00 |
| rs10195252 | 1237 | 0.864 | -94.4665 | 96.1945 | 0.986 | 1.00 |
| rs10508503 | 1233 | -29.9105 | -194.215 | 134.3935 | 0.721 | 1.00 |
| rs1055144 | 1244 | -64.3595 | -182.77 | 54.051 | 0.287 | 1.00 |
| rs10838738 | 1240 | -23.467 | -125.226 | 78.292 | 0.651 | 1.00 |
| rs10938397 | 1235 | 43.736 | -53.6235 | 141.095 | 0.379 | 1.00 |
| rs10968576 | 1244 | -19.764 | -121.848 | 82.3195 | 0.704 | 1.00 |
| rs1121980 | 1243 | 14.5635 | -78.6985 | 107.8255 | 0.760 | 1.00 |
| rs11847697 | 1245 | 236.988 | -46.41 | 520.3855 | 0.101 | 1.00 |
| rs12444979 | 1238 | 54.0695 | -95.143 | 203.2825 | 0.478 | 1.00 |
| rs1294421 | 1250 | -22.569 | -130.443 | 85.305 | 0.682 | 1.00 |
| rs12970134 | 1227 | -22.497 | -134.517 | 89.5225 | 0.694 | 1.00 |
| rs13107325 | 1247 | -143.375 | -405.51 | 118.76 | 0.284 | 1.00 |
| rs1424233 | 1229 | -31.318 | -130.369 | 67.733 | 0.536 | 1.00 |
| rs1443512 | 1249 | -104.597 | -219.4 | 10.207 | 0.074 | 1.00 |
| rs1514175 | 1242 | -29.9805 | -125.44 | 65.479 | 0.538 | 1.00 |
| rs1555543 | 1249 | -16.1485 | -118.08 | 85.783 | 0.756 | 1.00 |
| rs17782313 | 1242 | -89.8145 | -201.673 | 22.0435 | 0.116 | 1.00 |
| rs1805081 | 1230 | -78.4735 | -180.58 | 23.632 | 0.132 | 1.00 |
| rs206936 | 1248 | -46.3105 | -162.758 | 70.1365 | 0.436 | 1.00 |
| rs2112347 | 1234 | 12.8485 | -88.109 | 113.8065 | 0.803 | 1.00 |
| rs2241423 | 1236 | 11.0375 | -106.621 | 128.6955 | 0.854 | 1.00 |
| rs2287019 | 1242 | 44.044 | -76.4065 | 164.4945 | 0.474 | 1.00 |
| rs2568958 | 1238 | -167.249 | -266.054 | -68.443 | 0.001 | 0.045 |
| rs2605100 | 1249 | 2.7955 | -95.3345 | 100.9255 | 0.956 | 1.00 |
| rs2890652 | 1250 | -158.931 | -279.92 | -37.9415 | 0.010 | 0.502 |
| rs29941 | 1235 | -21.623 | -130.271 | 87.0245 | 0.697 | 1.00 |
| rs3810291 | 1231 | 7.996 | -92.5475 | 108.5395 | 0.876 | 1.00 |
| rs4712652 | 1230 | 10.687 | -85.447 | 106.821 | 0.828 | 1.00 |
| rs4771122 | 1233 | -25.171 | -130.431 | 80.089 | 0.639 | 1.00 |
| rs4823006 | 1245 | 63.954 | -31.351 | 159.2595 | 0.188 | 1.00 |
| rs4929949 | 1244 | -9.825 | -113.119 | 93.4695 | 0.852 | 1.00 |
| rs543874 | 1246 | 8.587 | -109.235 | 126.409 | 0.886 | 1.00 |
| rs545854 | 1245 | -97.189 | -221.344 | 26.9665 | 0.125 | 1.00 |
| rs6013029 | 1241 | -154.972 | -388.462 | 78.5185 | 0.193 | 1.00 |
| rs6232 | 1238 | -57.496 | -244.406 | 129.414 | 0.547 | 1.00 |
| rs6602024 | 1239 | -69.5325 | -237.984 | 98.919 | 0.419 | 1.00 |
| rs6784615 | 1252 | -137.688 | -356.244 | 80.8675 | 0.217 | 1.00 |
| rs6795735 | 1235 | 23.3205 | -74.399 | 121.0405 | 0.640 | 1.00 |
| rs6861681 | 1236 | 10.256 | -88.8935 | 109.4055 | 0.839 | 1.00 |
| rs6905288 | 1238 | -57.899 | -153.038 | 37.24 | 0.233 | 1.00 |
| rs713586 | 1249 | -20.135 | -118.535 | 78.265 | 0.688 | 1.00 |
| rs7138803 | 1242 | 3.0695 | -93.25 | 99.389 | 0.950 | 1.00 |
| rs718314 | 1234 | -20.3315 | -126.495 | 85.8315 | 0.707 | 1.00 |
| rs7647305 | 1236 | -87.192 | -208.643 | 34.259 | 0.159 | 1.00 |
| rs9491696 | 1239 | 81.544 | -18.183 | 181.271 | 0.109 | 1.00 |
| rs984222 | 1246 | 27.448 | -73.1995 | 128.096 | 0.593 | 1.00 |
| rs987237 | 1243 | -45.497 | -177.765 | 86.7705 | 0.500 | 1.00 |
| rs9939609 | 1240 | -2.914 | -98.4195 | 92.5915 | 0.952 | 1.00 |

**Table S2J**

| MONICA cohort,  Change in BW (gram/y/5 E% protein/risk allele). Protein replacing fat | | | | | | |
| --- | --- | --- | --- | --- | --- | --- |
| SNP | N | β | 95% CI | | p-value | corrected p-value |
| rs1011731 | 1245 | -57.9525 | -148.006 | 32.101 | 0.207 | 1.00 |
| rs10146997 | 1252 | 34.5885 | -76.29 | 145.467 | 0.541 | 1.00 |
| rs10195252 | 1237 | -28.19 | -119.55 | 63.1695 | 0.545 | 1.00 |
| rs10508503 | 1233 | -104.292 | -283.164 | 74.5805 | 0.253 | 1.00 |
| rs1055144 | 1244 | -41.4715 | -163.146 | 80.202 | 0.504 | 1.00 |
| rs10838738 | 1240 | 40.1345 | -55.166 | 135.4355 | 0.409 | 1.00 |
| rs10938397 | 1235 | -4.5955 | -96.168 | 86.977 | 0.922 | 1.00 |
| rs10968576 | 1244 | 74.936 | -23.9105 | 173.7825 | 0.137 | 1.00 |
| rs1121980 | 1243 | -64.714 | -152.271 | 22.842 | 0.147 | 1.00 |
| rs11847697 | 1245 | -158.026 | -393.578 | 77.526 | 0.189 | 1.00 |
| rs12444979 | 1238 | -107.53 | -242.885 | 27.8255 | 0.120 | 1.00 |
| rs1294421 | 1250 | -4.9385 | -104.098 | 94.2205 | 0.922 | 1.00 |
| rs12970134 | 1227 | -82.033 | -181.164 | 17.0975 | 0.105 | 1.00 |
| rs13107325 | 1247 | -125.958 | -321.569 | 69.6525 | 0.207 | 1.00 |
| rs1424233 | 1229 | 10.0625 | -85.3645 | 105.4895 | 0.836 | 1.00 |
| rs1443512 | 1249 | 42.955 | -62.0895 | 148 | 0.423 | 1.00 |
| rs1514175 | 1242 | 2.1435 | -91.119 | 95.406 | 0.964 | 1.00 |
| rs1555543 | 1249 | 75.892 | -16.078 | 167.8625 | 0.106 | 1.00 |
| rs17782313 | 1242 | -93.156 | -192.8 | 6.488 | 0.067 | 1.00 |
| rs1805081 | 1230 | -39.488 | -128.85 | 49.8735 | 0.386 | 1.00 |
| rs206936 | 1248 | -16.292 | -119.081 | 86.4975 | 0.756 | 1.00 |
| rs2112347 | 1234 | 21.9915 | -67.2605 | 111.2435 | 0.629 | 1.00 |
| rs2241423 | 1236 | 37.678 | -72.2545 | 147.61 | 0.502 | 1.00 |
| rs2287019 | 1242 | -55.991 | -167.68 | 55.697 | 0.326 | 1.00 |
| rs2568958 | 1238 | 2.9725 | -87.8435 | 93.789 | 0.949 | 1.00 |
| rs2605100 | 1249 | 23.516 | -70.402 | 117.434 | 0.624 | 1.00 |
| rs2890652 | 1250 | 4.658 | -110.238 | 119.553 | 0.937 | 1.00 |
| rs29941 | 1235 | -32.945 | -126.338 | 60.4475 | 0.489 | 1.00 |
| rs3810291 | 1231 | -36.3235 | -129.023 | 56.3755 | 0.443 | 1.00 |
| rs4712652 | 1230 | -72.346 | -160.706 | 16.0135 | 0.109 | 1.00 |
| rs4771122 | 1233 | -75.4755 | -172.494 | 21.5425 | 0.127 | 1.00 |
| rs4823006 | 1245 | -14.79 | -102.414 | 72.833 | 0.741 | 1.00 |
| rs4929949 | 1244 | 6.07 | -82.365 | 94.505 | 0.893 | 1.00 |
| rs543874 | 1246 | 24.518 | -78.5575 | 127.5935 | 0.641 | 1.00 |
| rs545854 | 1245 | -20.2645 | -145.113 | 104.5845 | 0.750 | 1.00 |
| rs6013029 | 1241 | 53.581 | -164.753 | 271.9155 | 0.631 | 1.00 |
| rs6232 | 1238 | -0.6485 | -180.454 | 179.157 | 0.994 | 1.00 |
| rs6602024 | 1239 | -76.4425 | -225.862 | 72.977 | 0.316 | 1.00 |
| rs6784615 | 1252 | 63.8235 | -161.512 | 289.159 | 0.579 | 1.00 |
| rs6795735 | 1235 | 58.0965 | -35.315 | 151.508 | 0.223 | 1.00 |
| rs6861681 | 1236 | 99.948 | 9.311 | 190.585 | 0.031 | 1.00 |
| rs6905288 | 1238 | -15.324 | -101.232 | 70.584 | 0.727 | 1.00 |
| rs713586 | 1249 | 12.205 | -80.197 | 104.607 | 0.796 | 1.00 |
| rs7138803 | 1242 | 56.4035 | -32.6615 | 145.4685 | 0.215 | 1.00 |
| rs718314 | 1234 | 10.929 | -88.16 | 110.0175 | 0.829 | 1.00 |
| rs7647305 | 1236 | 106.181 | -8.343 | 220.7045 | 0.069 | 1.00 |
| rs9491696 | 1239 | 49.7985 | -42.732 | 142.329 | 0.292 | 1.00 |
| rs984222 | 1246 | -83.5505 | -179.21 | 12.1085 | 0.087 | 1.00 |
| rs987237 | 1243 | -13.2155 | -130.249 | 103.8175 | 0.825 | 1.00 |
| rs9939609 | 1240 | -72.0285 | -163.081 | 19.024 | 0.121 | 1.00 |

**Table S3**: Protein intake within energy balance and BMI SNP-score level.

|  |  |  | **Protein E%** | | | | | **Protein gram/kg body weight/day** | | | | |
| --- | --- | --- | --- | --- | --- | --- | --- | --- | --- | --- | --- | --- |
| Cohort | Energy balance | BMI SNP-score | N | Mean | SEM | 95 % CI | | N | Mean | SEM | 95 % CI | |
| MONICA | Negative | Low | 77 | 14.19 | 0.30 | 13.59 | 14.78 | 77 | 1.084 | 0.04 | 1.01 | 1.16 |
|  |  | High | 47 | 15.41 | 0.51 | 14.39 | 16.43 | 47 | 0.97 | 0.05 | 0.88 | 1.07 |
|  | Neutral | Low | 291 | 13.97 | 0.16 | 13.65 | 14.29 | 291 | 1.17 | 0.02 | 1.13 | 1.21 |
|  |  | High | 189 | 14.20 | 0.21 | 13.78 | 14.62 | 189 | 1.10 | 0.02 | 1.05 | 1.15 |
|  | Positive | Low | 194 | 14.94 | 0.23 | 14.50 | 15.39 | 194 | 1.11 | 0.02 | 1.07 | 1.15 |
|  |  | High | 143 | 14.28 | 0.25 | 13.78 | 14.77 | 143 | 1.10 | 0.04 | 1.03 | 1.17 |
| DCH | Negative | Low | 86 | 17.70 | 0.28 | 17.14 | 18.26 | 86 | 1.25 | 0.04 | 1.17 | 1.33 |
|  |  | High | 95 | 18.04 | 0.27 | 17.50 | 18.57 | 95 | 1.24 | 0.04 | 1.16 | 1.32 |
|  | Neutral | Low | 232 | 17.59 | 0.16 | 17.27 | 17.90 | 232 | 1.39 | 0.03 | 1.34 | 1.44 |
|  |  | High | 194 | 17.56 | 0.18 | 17.20 | 17.91 | 194 | 1.31 | 0.03 | 1.25 | 1.37 |
|  | Positive | Low | 407 | 17.82 | 0.13 | 17.57 | 18.08 | 407 | 1.24 | 0.02 | 1.20 | 1.28 |
|  |  | High | 424 | 17.85 | 0.14 | 17.58 | 18.12 | 424 | 1.20 | 0.02 | 1.16 | 1.23 |
| INTER99 | Negative | Low | 248 | 15.11 | 0.15 | 14.82 | 15.39 | 248 | 1.02 | 0.02 | 0.97 | 1.06 |
|  |  | High | 163 | 15.12 | 0.18 | 14.76 | 15.48 | 163 | 1.00 | 0.03 | 0.94 | 1.05 |
|  | Neutral | Low | 642 | 14.94 | 0.09 | 14.76 | 15.11 | 642 | 1.20 | 0.02 | 1.17 | 1.23 |
|  |  | High | 430 | 14.96 | 0.11 | 14.74 | 15.18 | 430 | 1.20 | 0.02 | 1.15 | 1.25 |
|  | Positive | Low | 449 | 15.02 | 0.11 | 14.80 | 15.24 | 449 | 1.15 | 0.02 | 1.12 | 1.19 |
|  |  | High | 290 | 15.10 | 0.16 | 14.79 | 15.41 | 290 | 1.14 | 0.02 | 1.10 | 1.19 |

Energy balance was decided from average weight change during the study period. Negative energy balance: average weight loss of >0.5 kg/year. Neutral energy balance: average weight change of +/- 0.5 kg/year. Positive energy balance: average weight gain of >0.5 kg/year. BMI SNP-score: above or below the mean score.
